# COVID-19 and the scientific publishing system: growth, open access and scientific fields

**DOI:** 10.1007/s11192-022-04536-x

**Published:** 2022-10-10

**Authors:** Gabriela F. Nane, Nicolas Robinson-Garcia, François van Schalkwyk, Daniel Torres-Salinas

**Affiliations:** 1 Delft Institute of Applied Mathematics (DIAM), Delft University of Technology, Delft, Netherlands; 2 EC3 Research Group, Information and Communication Studies Department, University of Granada, Granada, Spain; 3 DST-NRF Centre of Excellence in Scientometrics and Science, Technology and Innovation Policy, Centre for Research on Evaluation, Science and Technology, Stellenbosch University, Stellenbosch, South Africa

**Keywords:** COVID-19, Scientific publications, Growth of science, Dimensions, Open access

## Abstract

We model the growth of scientific literature related to COVID-19 and forecast the expected growth from 1 June 2021. Considering the significant scientific and financial efforts made by the research community to find solutions to end the COVID-19 pandemic, an unprecedented volume of scientific outputs is being produced. This questions the capacity of scientists, politicians and citizens to maintain infrastructure, digest content and take scientifically informed decisions. A crucial aspect is to make predictions to prepare for such a large corpus of scientific literature. Here we base our predictions on the Autoregressive Integrated Moving Average (ARIMA) and exponential smoothing models using the Dimensions database. This source has the particularity of including in the metadata information on the date in which papers were indexed. We present global predictions, plus predictions in three specific settings: by type of access (Open Access), by domain-specific repository (SSRN and MedRxiv) and by several research fields. We conclude by discussing our findings.

**Supplementary Information:**

The online version contains supplementary material available at 10.1007/s11192-022-04536-x.

## Introduction

The average growth in scientific publications is estimated to be 4% per annum, doubling approximately every 17 years (Bornmann et al., 2021). And for developing countries total growth is estimated to be 8.6% (National Science Foundation, 2018). Unsurprisingly, and given the scale of scientific output, one of the main research topics within the field of scientometrics has been the study of the growth of scientific literature. Indeed, in the 1960s, Derek de Solla Price (1963) had already developed a model of the exponential growth of science in what is one of the seminal contributions to the field. Although his study was not the first attempt to model the growth of scientific literature (Coles & Eales, 1917; Hulme, 1923), it reflects the predominant role that the study of bibliometric distributions, dynamics of growth and ageing laws of scientific literature has had in the field.

According to Price's model, there are three distinct phases by which scientific literature increases over time. In the first phase, there is a slow incremental increase in publications, followed by an exponential increase, and then a third phase in which the growth curve reaches a saturation point. Different studies have tried to refine his approach by trying to identify mathematical models that can accurately adjust growth curves for the observed increase in scientific literature. These studies reflect continued efforts to identify distributions and models which can best adjust to different types of scientific literature in different conditions. In general terms, these studies describe science as a complex ecosystem (van Raan, 2000), which grows exponentially, and is sensitive to social, political and economic developments (Bornmann et al., 2021), following differing patterns by field (Egghe & Rao, 1992).

The most recent social development which has affected the production of new scientific knowledge globally is the COVID-19 pandemic. The outbreak of the pandemic early in 2020 led to an unprecedented surge in scientific publications in an effort to share as much new knowledge as rapidly as possible to find scientifically sound solutions to end or at least curtail the spread of the coronavirus (Brainard, 2021). The demand for solutions and the resultant surge in the number of covid-related publications has introduced new pressures while simultaneously creating new opportunities in the scholarly publishing system (Brainard, 2020; Brinton, 2021; Kupferschmidt, 2020; O’Connor, 2021; Watkinson, 2021).

In this paper we investigate the growth of scientific literature related to COVID-19 in the exceptional circumstance of the coronavirus pandemic. This unique phenomenon disrupted the scientific knowledge production system, mobilizing researchers from all scientific fields across the world. Such mobilization could be observed on the allocation of scholarly efforts (Haghani & Bliemer, 2020), changes in collaboration patterns (Cai et al., 2021), and changes in the publishing system (Palayew et al., 2020). A call for promoting Open Access was made and responded by all main publishers, who opened the access to their contents (Tavernier, 2020), and authors turned to pre-printing, in order to accelerate the publication of their findings (Fraser et al., 2021). Furthermore, there is an interest to investigate whether the pandemic influenced the growth of scientific literature disproportionately by research field. The focus on health related research has been complemented, later on in the pandemic, by the focus on the economic or other social societal aspects, such as education. We build our conceptual framework upon existing literature on scientific growth, and we question to what extent the production of COVID-19 related studies has followed similar growth patterns.

The scientometric community has made an effort to describe the contents of new data sources liberated specifically on the topic of COVID-19 (Colavizza et al., 2021), to compare the coverage of different data sources (Kousha & Thelwall, 2020; Torres-Salinas et al., 2021), to analyze the effectiveness of scholarly communication in these pressing times (Homolak et al., 2020; Soltani & Patini, 2020), and to understand its consumption in social media (Colavizza et al., 2021; Nane et al., 2021; Thelwall, 2020). The present study is integrated within this stream of literature, building on preliminary findings (Torres-Salinas et al., 2020). This paper is a substantially reworked version of (Torres-Salinas et al., 2021), with new data, updated analysis and discussion.

It aims to forecast the growth of COVID-19 literature to better understand the magnitude of the phenomenon a year after the outbreak of the pandemic and to provide early insights into the extent to which it has disrupted the scientific publishing system. The rapid and overwhelming response of the scientific community to the COVID-19 pandemic led many to suggest that it would transform the scholarly communication system (Brainard, 2020, 2021; Larivière et al., 2020). Specifically, we aim at addressing the following research questions:

- *RQ1* Does growth in COVID-19 science during the pandemic follow the same patterns as scientific knowledge during ‘normal’ times?
- *RQ2a* Do we observe a shift in the scientific publishing system towards open access during the pandemic?
- *RQ2b* Are green open access articles gaining greater momentum compared with gold open access journal articles during the COVID-19 pandemic?
- *RQ3a* After more than a year of the pandemic, do we observe differences in growth rates between scientific fields?
- *RQ3b* How will these differences (if any) evolve over time?

Along with modelling growth curves to specific scientific output, more general forecast methods can and have been employed. Historical data can be recorded as a time series, where output is registered at successive equally spaced points in time. For example, Taşkın (2021) provides a review of time series forecasting studies in library and information science. Forecasting trends were also investigated to study different fields; for example, in psychology, exponential smoothing was employed in Krampen et al. (2011). The main difference with the growth models lies in the lack of typical parametric model families for the time series forecasting methods, where trends or growth are modelled using more flexible models.

To respond the research questions, we have structured the paper as follows. First, we investigate how COVID-19 research has affected the scientific publishing system. From the emergence of new databases devoted to COVID-19 literature, to the response by the research community, funding agencies and scientific publishers. We review studies analyzing changes in the publishing behavior of academics and how these changes have affected scientific literature consumption and production. We then describe the methodological design followed in this paper. We describe the data used, as well as the development of different sub datasets directed at responding to each of our research questions. We report our findings by first fitting different growth and prediction models to our data. We validate the models using measures that indicate predictive performance. Choosing the best predictive models, we report prediction of growth for each of the sub datasets created. We conclude by discussing how these findings fit into the expectations articulated in previous studies related to major changes in the scientific publishing system.

## The impact of the pandemic on scholarly publishing: a review of the literature

The Covid-19 pandemic has placed unprecedented pressure on science to respond to and combat a global crisis. Scientists have put on hold their research agendas to redirect their focus towards the disease and its effects in society (Gibney, 2020). Funding agencies have developed new funding streams (Kaiser, 2020) and journals have accelerated their peer review processes to rapidly make COVID-19-related research public (Horbach, 2021). The mobilization of resources and human capital has been such that the growth in COVID-19 literature was already significant just 1 month after the World Health Organization declared COVID-19 a world pandemic (Torres-Salinas, 2020).

However, growth numbers differed depending on the database used. While Scopus reported around 12,000 papers in June 2020 (Zyoud & Al-Jabi, 2020), this number had already been reached a month earlier according to the Dimensions database (Torres-Salinas et al., 2020). In fact, researchers were uploading preprints in repositories without waiting for journal publication or even acceptance (Brainard, 2020), making it a challenge to manage the amount of scientific literature related to COVID-19. In parallel, different initiatives took off to develop special collections of COVID-19 literature, the most notable being the CORD-19 dataset (Colavizza et al., 2021) and the World Health Organization COVID-19 Database (WHO, 2020).

The surge of COVID-19 papers brought into question how the scholarly communication system operates (Kupferschmidt, 2020; Larivière et al., 2020). The pace of peer review and scientific publishing in scientific journals did not match the urgent necessity to share findings and accelerate discovery to put an end to the pandemic. This led some researchers to turn to open access publication by uploading their research directly to preprint servers and repositories without going through a formal peer review process (Callaway, 2020; Zastrow, 2020). This resulted in an increase in the number of preprints and the attention they received (Fraser et al., 2021). With the general state of emergency, it was not only scientists who were looking for answers; journalists and non-experts were equally attentive to claimed advances in our understanding of the virus (Gulbrandsen et al., 2020), resulting in one of the biggest disruptions to the certification process in the formal science communication system (Chiarelli et al., 2019; Sohrabi et al., 2021).

By early 2021, with over half a million COVID-19-related papers published, claims that COVID had overhauled the scientific publishing system remained inconclusive (Brainard, 2021; Grant, 2021). This is partly explained by the quick response of publishers. Indeed, while COVID-19 preprints increased, so did journal articles (Fraser et al., 2021). A study comprising 14 medical journals found that journals had accelerated their publication processes; the time between submission and the publication of the journal articles decreased on average by 49% (Horbach, 2020). A follow-up study reported that journals had lowered their quality standards to reduce the timing from submission to publication (Horbach, 2021), which could explain a number of retractions (Soltani & Patini, 2020).

But publishers’ response has not only been limited to speeding up publication processes; they have also opened their content, albeit temporarily (Gadd, 2020), suggesting a move towards open access. However, publishers have not always liberated their copyright licenses and much of the current open access literature can be described as ‘bronze’ open access (Torres-Salinas et al., 2020), that is, free-to-read articles made available by publishers without an explicit mention of open access (Robinson-Garcia et al., 2020).

## Data and methods

### Data collection and processing

In June 2021, we downloaded the COVID-19 dataset provided by Dimensions (2020). This dataset was created in March 2020 and is updated periodically. It includes four sub- datasets (publications, clinical trials, grants, and datasets indexed in the Dimensions database), and was retrieved using the following query in the full text for publications from 2020:

“2019-nCoV” OR “COVID-19” OR “SARS-CoV-2” OR “HCoV-2019” OR “hcov” OR “NCOVID-19” OR “severe acute respiratory syndrome coronavirus 2” OR “severe acute respiratory syndrome corona virus 2” OR “coronavirus disease 2019” OR ((“coronavirus” OR “corona virus”) AND (Wuhan OR China OR novel))

We emphasize that we consider both versions of “corona virus” and “coronavirus”, in order to include as many publications possible. In this paper we worked with version 40 of the dataset, which covers the period January 1st, 2020, to May 31st, 2021. We only include publications in our analysis, comprising a total of 464,217 records. For each record, this database includes the date when the record was added to the database, publication source (including journals, repositories and books and conference proceedings), document type (article, preprint, proceeding, chapter, book and monograph^1^), and the open access status of the record, along with other bibliographic metadata. These three characteristics make this database unique for our purposes. While the WHO database has similar characteristics, it has a lower coverage than Dimensions (Torres-Salinas et al., 2021).

The date when records were added is a key field for our study, as it includes the day, month and year when a paper was first registered in Dimensions. This information differs from the publication date, as papers may be available online as early access prior to being assigned to a journal issue. This field was used to model and make predictions on the growth of publications.

To answer our research questions, we grouped our data into different subsets. These subsets allowed us to address specific growth patterns with regard to open access (OA) versus non-open access (non-OA) literature; green versus gold open access; and different research fields. Dimensions classifies access to documents based on data derived from Unpaywall (Porter & Hook, 2020). Unpaywall is a service which offers access to open versions of scholarly literature (Piwowar et al., 2018). It distinguishes between seven types of access: closed; all OA, Bronze; all OA, Green, submitted; all OA, Green, published; all OA Green, accepted; all OA, pure Gold; and, all OA, Hybrid. Dimensions does not clearly define how these types are identified or defined (Porter & Hook, 2020), and the categories are presented as exclusive, meaning that green open access is probably underrepresented (Robinson-Garcia et al., 2020).

The first two time series collect OA or non-OA records. The purpose of analyzing these subsets is to find differences in terms of growth by type of access in relation to COVID-19 literature. The second time series distinguishes between gold and green only OA. These time series include all OA, green, submitted and all OA, green accepted. In this case, published green OA documents are excluded to avoid double counting preprints which end up published in OA journals.

The last time series are based on the field of research of the records. The COVID-19 dataset does not provide information about the field of research. However, this information is available in the Dimensions database. We matched our dataset with Dimensions and extracted the field of research assigned to each record. Dimensions uses the Australian and New Zealand Standard Research Classification, which is a three-level classification. We worked with the 22 Fields of Research (FoR). Although the Dimensions classification system has been criticized for its use of machine learning and its inaccuracy (Bornmann, 2018), these comments are based on lower levels of the classification and on anecdotal evidence. In the case of records stored in repositories, field-specific repositories were manually assigned to a field (e.g., MedRxiv was assigned to the field Medical & Health Sciences). When this was not possible, publications were left unassigned—16.4% of the records were not assigned to any field. In this case, we only analyzed the five largest fields, which represent 66.2% of the complete dataset. Table 1 shows an overview of the total number of records per timeseries.

**Table 1:** Overview of time series and corresponding number of publications

	Timeseries	No. of records
Type of Access	Open Access	337,906
	Non Open Access	126,209
OA uptake	Gold Open Access	288,246
	Green Open Access (only)	22,760
Fields	Medical & Health Sciences	229,110
	Biological Sciences	26,071
	Studies in Human Society	19,782
	Economics	18,052
	Education	14,143
	All publications	464,115

### Forecasting models

We first aimed to fit standard growth models, such as exponential or logistic growth models, which have typically been employed to model scientific output growth (see, for example, Bornmann et al., 2021; Egghe & Rao, 1992). The two growth models did not fit the data well enough for them to be used for forecasting. The plots illustrating the fitting are included in the Supplementary material (Part 1). The plots clearly indicate that none of the time series follow an exponential growth. Even though most of the time series follow logistic growth reasonably well, this growth model fails to fit the most recent observations, therefore missing the increasing trend in the most recent part of the data. For logistic regression, uncertainty bounds were computed from the uncertainty in the estimated parameters of the model. However, the uncertainty bounds are very narrow for the beginning and end of the timeseries, which is an undesired behavior. Moreover, for predictions, it was not possible to compute uncertainty bounds. We consider this a significant disadvantage, as we are interested not only in point forecasts, but also in predicting a range of possible forecasts.

For these reasons, we use well-established statistical methods to model and forecast time series historical data of publication growth. Two classes of methods stand out for time series forecasting: Auto Regressive Integrated Moving Average (ARIMA), and exponential smoothing models (Hyndman & Athanasopoulos, 2018). ARIMA(*p,d,q*) models employ differencing the time series to ensure that the stationarity assumption holds. Intuitively, stationarity entails that the properties of the time series, such as the average or variance, do not vary with time. The necessary number of differencing to ensure stationarity is indicated by the parameter *d*. Furthermore, forecasts are modelled as a linear combination of past observations and past error terms of the model. The number of past observations is denoted by the parameter *p*, whereas the number of past error terms is denoted by the parameter *q*.

The three parameters are estimated using the *auto.arima* function in the forecast R package, which is based on a variation of the Hyndman-Khandakar algorithm (Hyndman & Khandakar, 2008). The procedure starts by estimating parameter *d*, using stationary hypothesis testing. Then parameters *p* and *q* are estimated using the corrected Akaike’s Information Criterion (AIC). Finally, once the order of the ARIMA model is identified, the coefficients of the past observations and errors are estimated using a maximum likelihood procedure.

Exponential smoothing (ES) methods yield forecasts based on exponentially decaying weighted averages of past observations. This class of methods does not require the stationarity assumption and can hence model the trend of the data. The model uses smoothing parameters and initial states, which are estimated from data by maximum likelihood.

Both ARIMA and ES are established methods that have been shown to perform well in forecasting time series data. While ARIMA focuses on the stationarity assumption and attempts to reach this by performing various transformations of the data, ES methods do not require stationarity. Moreover, ARIMA accounts for error terms at previous times steps, whereas ES only models data at previous time points.

Both ARIMA and ES models were fitted to the time series considered for our analysis. All the analyses were conducted on a Windows 10 machine, running R version 4.0.5 and RStudio version 1.4.1106. Point forecasts are complemented by 95% prediction intervals. Data are available at 10.5281/zenodo.5774870, while supplementary material with results and analyses of this study are openly accessible at 10.5281/zenodo.5774878.

The models were evaluated with respect to how well they fit the data and to the predicted performance. The mean absolute deviation (MAD) is used as a performance measure. MAD is the average of the absolute differences between the model’s estimations and the observations. It is also known as the mean absolute error (MAE). The models were validated on prediction performance using a training and a testing set. The training set included approximately 80% of the data, and the test set comprised approximately 20% of the data. The training set consisted of data from January 1st, 2020, up to and including February 17th, 2021, whereas the test set accounted for data from February 18th to May 31st, 2021. MAD for the training set therefore accounted for how accurately the models fit the data, whereas MAD for the test set reported the prediction accuracy of the models.

MAD enables the comparison between the two forecast models, ARIMA and ES, for each time series. The best predictive model was used to make the forecasts for the time series. Note that the MAD values depend on the scale of the time series. In order to compare model performance across time series, a scale-independent measure should be used. We employ the mean absolute percentage error (MAPE), given by ${\text{mean}}\left( {\left| {100\left( {y_{t} - \hat{y}_{t} } \right)/y_{t} } \right|} \right)$, the average of percentage errors, which are calculated by differencing the time series observations $y_{t}$ with the model’s predictions $\hat{y}_{t}$, scaled by the observations $y_{t}$.

Along with point forecasts, models report 95% prediction intervals, to account for the uncertainty encompassing the point estimates. In the forecast package in R, prediction intervals are calculated based on the assumption that the forecast errors are normally distributed. Moreover, the prediction intervals increase as the forecast horizon increases, based on the assumption that more uncertainty is inherited by forecasts further in the future. The increase is given by the time step of the forecast and estimated residual standard deviation of the corresponding forecast distribution.

We consider two performance measures to account for the uncertainty of the predictions, the average length (AL) and the coverage percentage (CP) of the 95% prediction intervals. AL gives the average of all prediction intervals’ length in the test set, and CP computes the percentage of data in the test set that lies within (is covered) by the prediction intervals. Intuitively, AL gives information about how informative the prediction intervals are, whereas CP reveals how well the prediction intervals capture the observations.

## Results

We present results of the forecast models for all the COVID-19 publications, and their distribution according to the type of access, and open access uptake. Also, we include results for the top five fields: Medical & Health Sciences; Biological Sciences; Economics; Studies in Human Society; and Education. We first validate the two forecasting models with respect to the predictive performance. Then the best predictive model is chosen for each time series of interest to predict the number of publications up until the end of March 2022.

### Validation of forecasting models

**Fig. 1:**
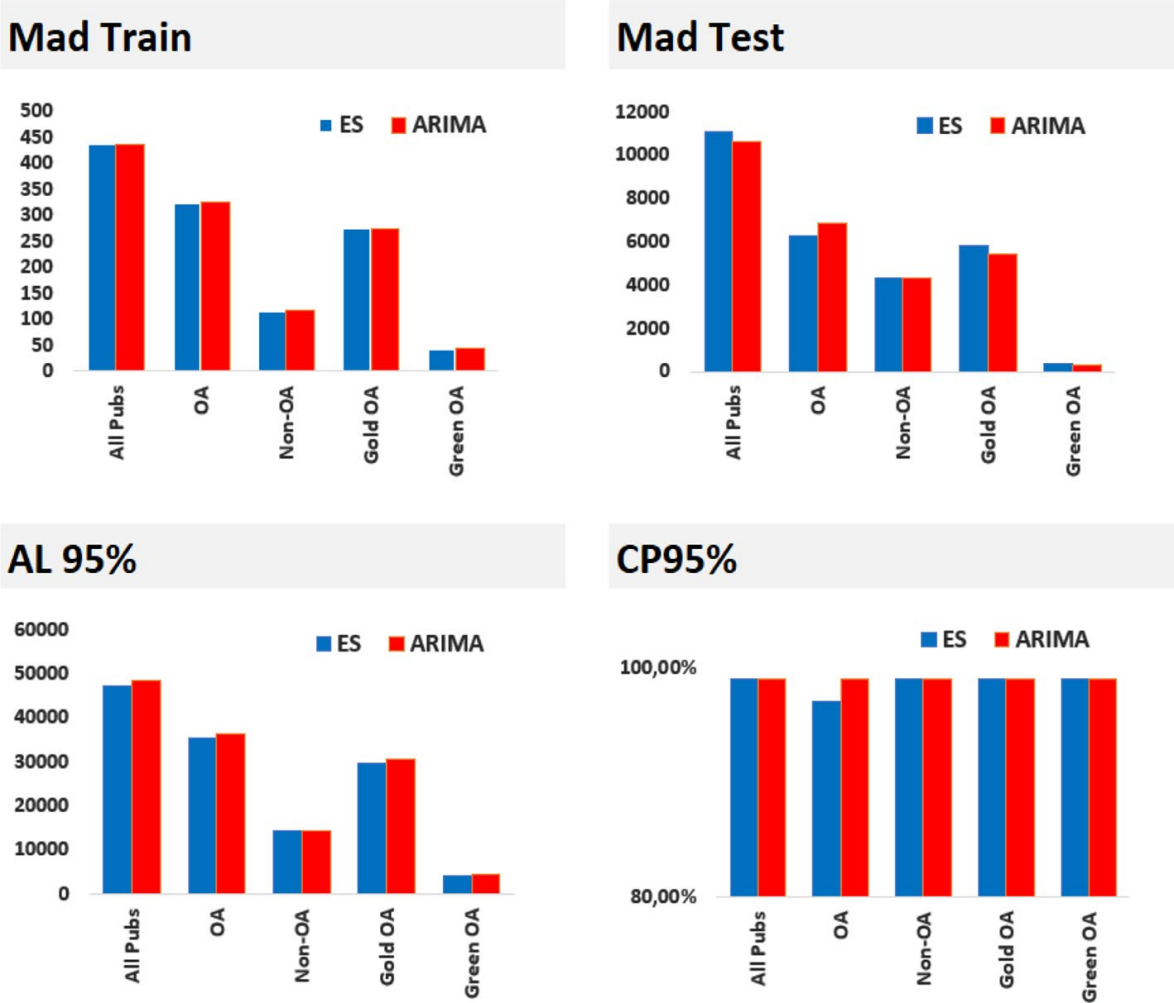
Model validations for complete time series, OA, non-OA publications, gold OA and green OA (only). Top figures include mean absolute deviations (MAD) for the training and test set for exponential smoothing (in blue) and ARIMA (in orange) models. The bottom figures show the average length (AL) and coverage probability (CP) of prediction intervals for exponential smoothing (blue) and ARIMA (red). (Color figure online)

We validate the models by investigating their predictive performance, using the mean absolute deviation (MAD), the average of absolute errors, calculated over the test set. The results for all the time series are depicted in Fig. 1(top right). The most notable difference is for all publications and Gold OA, where ARIMA outperforms ES. For OA publications, ES outperforms the ARIMA model. For the remaining two time series, the differences are minor. The very small MAD for green OA is mainly driven by the very small number of publications in comparison with the other time series. The MAD test results also bring information into the forecast error. First, it is notable that both models overestimate the number of publications, except for the first five forecasted time points for Gold OA. For all publications, the forecast error averages around 10,600 publications for ARIMA and 11,000 publications for ES. For Green OA, the forecast error is off, on average, by 320 publications for ARIMA and by 346 publications for ES.

Note that the MAD values should not be compared across time series, due to the scale differences, since the forecast error is dependent on the scale of the time series. We report the scale-independent mean absolute percentage error (MAPE) results for the best predictive model, in terms of MAD in the test set, of each time series. An average relative error of 2.29% is obtained for the ARIMA model for all publications, 1.98% for OA (ES) and 3.67% for Non-OA (ARIMA). For Green OA, a 1.32% mean absolute percentage error rate is obtained, whereas for Gold OA, the MAPE is 1.82%. For both time series, the ARIMA model has been employed. Overall, the models show a very good performance in terms of the error rate.

For comparison with MAD test, we include the MAD for the training set (Fig. 1, top left). This measure describes how well the models fit the data and contrasts the MAD for the test set by the fact that the first evaluates the fit of the data, whereas the latter evaluates prediction of new data. The first observation is that MAD in the training set are very small values, in comparison with test MAD values. The training set differences between the two models are modest. However, for OA, it seems that ES fits the data better, but ARIMA predicts better. Despite the small differences, this shows that fitting performance is not always a good indicator for predictive performance.

Along with point estimates, model forecasts also include uncertainty estimates. We consider the average length (AL, Fig. 1 bottom left) and the coverage probability (CP, Fig. 1 bottom right) of the 95% prediction intervals in the test set. It is noticeable that both models yield prediction intervals with an impressive coverage probability. The CP performance for Green OA and Non-OA is doubled by the AL performance; with small prediction intervals, on average, a very high coverage probability is achieved by both forecasting models.

The two measures usually have a positive correlation. That is, the higher AL, the higher CP. This holds for the OA data, where the slightly larger prediction intervals, on average, resulted in a higher coverage probability. Interestingly, that seems not to be the case for a few time series, though, again, the differences are small. For example, for all publications, exponential smoothing results in smaller prediction intervals, on average, whereas the coverage of the predictions remains the same.

**Fig. 2:**
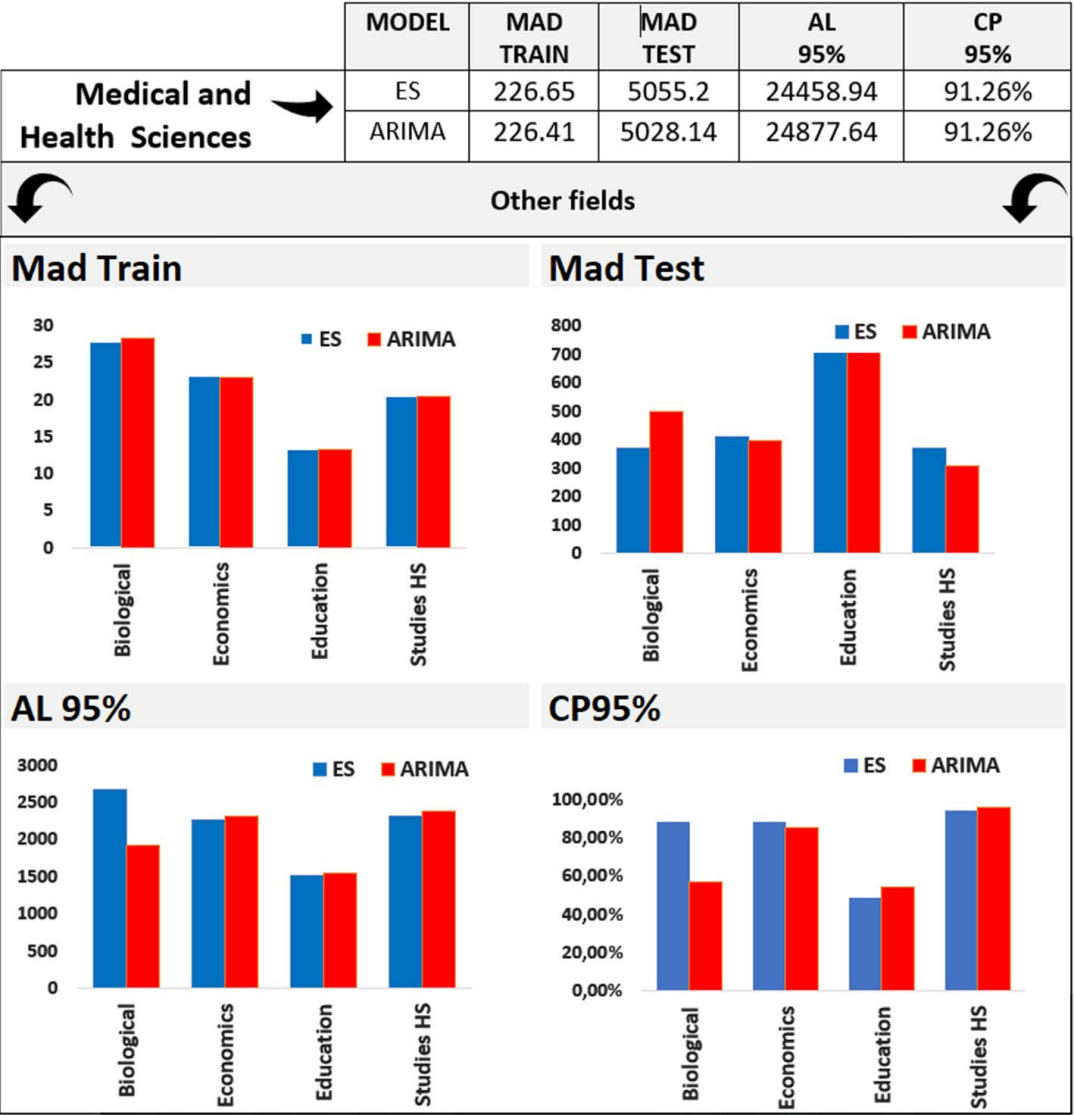
Model validations for time series by research field. Only the top five fields are included: Medical & Health Sciences, Biological Sciences, Economics, Studies in Human Society, and Education. Top figures include mean absolute deviations (MAD) for the training and test set for exponential smoothing (in blue) and ARIMA (in orange) models. The bottom figures show the average length (AL) and coverage probability (CP) of prediction intervals for exponential smoothing (blue) and ARIMA (red). (Color figure online)

We validated ES and ARIMA models for the most prolific fields in COVID-19 research topics. We again observe no dramatic differences between the performance of the two models. For Medical & Health Sciences, the largest field covering COVID-19 literature, ARIMA yields the lowest MAD in the test set, with smaller prediction intervals, on average and a high coverage probability (Fig. 2, table above).

For the remaining four fields, the most notable differences in the test set are registered for fields of Biological Sciences, where the ES prediction performance in the test set exceeds the performance in the test set, and Studies in Human Society, where ARIMA outperforms ES in terms of predictive performance, despite similar MADs in the training set. For Biological Sciences, ARIMA leads to overconfident prediction intervals, as shown by the smaller AL coupled with the modest CP. The two forecast models show good performance for all fields except Education, for which the highest MAD results are combined with the lowest CP. Except Medical & Health Sciences, all other fields have a comparable number of publications, as reported in Table 1. Nonetheless, we computed MAPE for all fields. The best MAPE is achieved by ES for Biological Sciences, with a 1.03% average relative error. It is followed by ARIMA model Studies in Human Society, with 1.14% average relative error. ARIMA models for Medical and Health Sciences and Economics yield a 1.93%, respectively 1.91% average relative errors. ES for Education records the poorest MAPE of 5.53%. The validation plots for all the models are included in the Supplementary material (Part 2).

### Predictions by time series

**Fig. 3:**
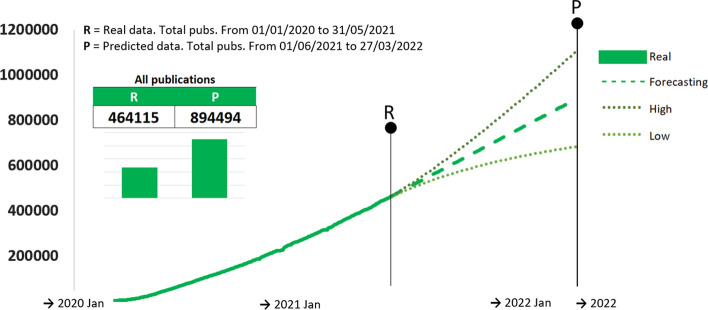
One-year predictions of the accumulated number of publications expected for all publications related to COVID-19 literature

We used the validation step to identify the best predictive model for each time series. This was chosen by using the MAD results in the test set, and can be identified from Figs. 1 and 2. We now use those models to make predictions until the end of March 2022. The point forecasts for all the COVID-19 publications, along with the 95% predictions are illustrated in Fig. 3.

We report the forecast for 27 March 2022, of 894,494 publications (denoted by P in Fig. 3), which is 1.93 times larger than the last recorded observation of 464,115 publications (denoted by R in Fig. 3). This indicates a significant expected increase over the next 10 months.

**Fig. 4:**
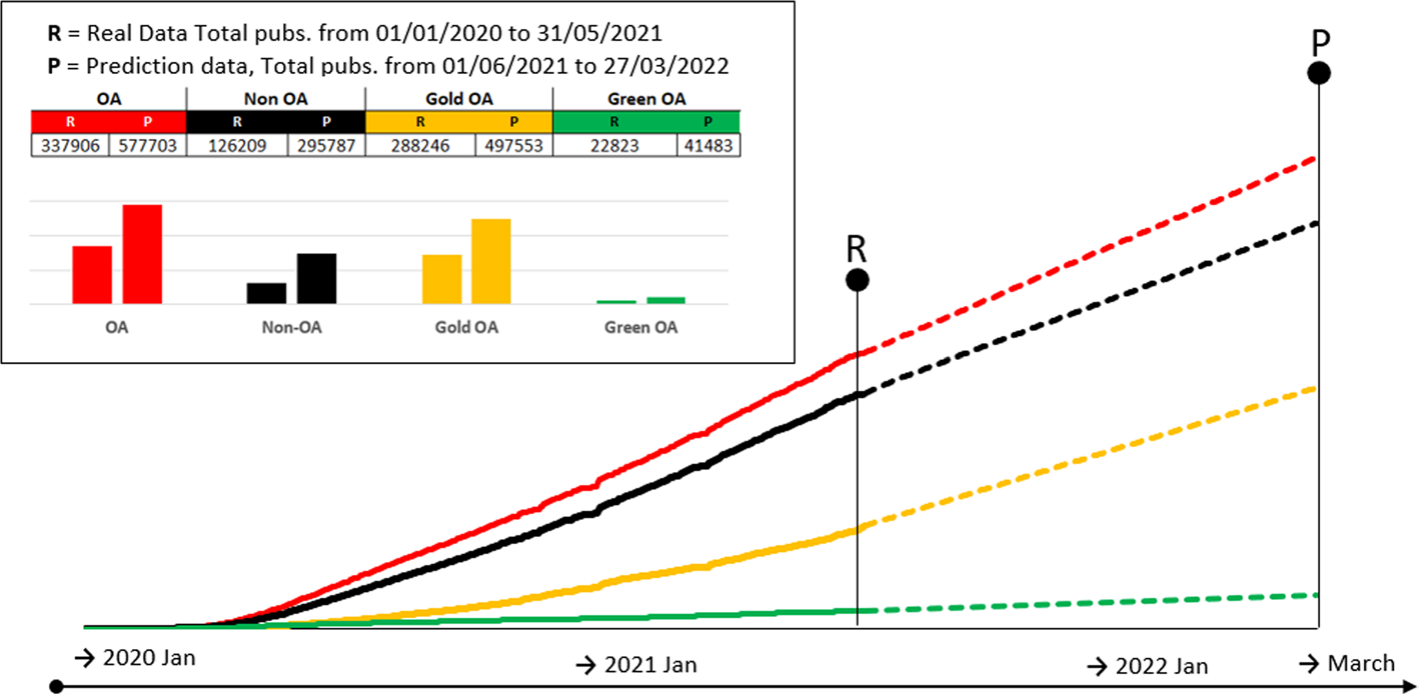
One-year predictions of the accumulated number of publications expected for OA publications (red), non-OA (black), Gold OA (yellow) and Green OA (green) related to COVID-19 literature. (Color figure online)

Figure 4 illustrates the forecasted growth in the number of publications according to document type. Both existing and forecasted OA and Non-OA publications maintain similar growth trends. Despite a slow start, Gold OA publications exhibit an increase in growth at a higher rate in late 2020 and early 2021. Green OA registers both the smallest number of publications, as well as the weakest growth over the period data were collected. The forecast maintains approximately the same level of growth.

**Fig. 5:**
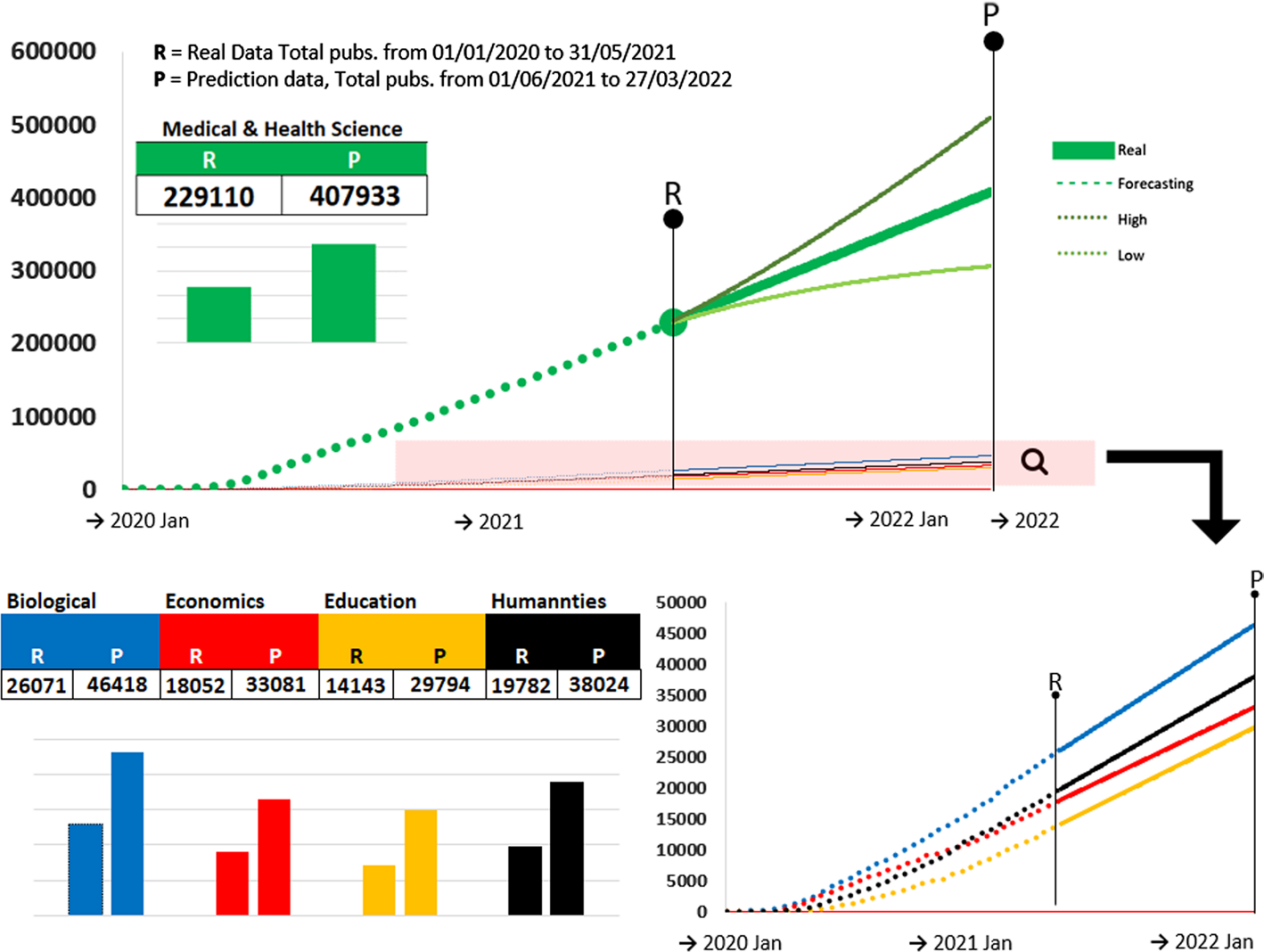
One-year predictions of the accumulated number of publications expected for publications related to COVID-19 literature in the fields of Medical & Health Sciences (green), Biological Sciences (blue), Economics (red), Studies on Human Society (yellow), and Education (black). (Color figure online)

Figure 5 depicts the forecasted growth for the five analyzed fields. The largest field, Medical & Health Sciences (Fig. 5, top), is expected to grow by a factor of 1.78 from the time publications were last collected (R) to the furthermost prediction time (P).

The forecasts for the other four fields (Fig. 5, bottom) exhibit similar growth patterns, except Economics (in red), which is expected to register a slight decrease in the number of publications by 27 March 2022. The differences need however to be quantified, as visual differences can be misleading. Biological Sciences is expected to grow by the same factor, of 1.78, as Medical & Health Sciences. Despite the apparent slower predicted growth, Economics is expected to grow by a factor of 1.83, which is slightly higher than the predicted growth of the other fields. The highest growth factor of 2.10 is registered by Education, whereas Study of Human Society has an expected growth factor of 1.92.

## Discussion

This study examined growth patterns of COVID-19 literature in order to understand the extent to which it has disrupted the scientific publishing system. To do so, we confronted publication data with different forecasting models and presented 1-year predictions of future growth for the COVID-19 literature overall, by type of access and by fields. Before going through the main findings of the study we note certain limitations. First, it is possible that the use of other datasets might make a difference to the modelling and forecasting results. Identifying the boundaries of COVID-19 related literature has become a challenge (Colavizza et al., 2021) with different data sources now available showcasing some differences between each other (Torres-Salinas et al., 2021).

Second, although framed within studies related to the growth of science, our analysis was focused on a relatively short time-period; when compared with other similar papers (e.g., Bornmann & Mutz, 2015); while they looked at years as units of analysis, we analyzed daily data. Similarly, times of crisis may well have unique impacts on scientific output (Zhang et al., 2020), as may anomalies within the crisis period (see below). All that we can say at this point is that these factors may account for differences without knowing the full impact that they may have on the forecasts presented in this paper.

The prediction for the number of scientific publications related to COVID-19 being published between 1 June 2021 and 27 March 2022 shows steady growth that does not deviate from the actual number of publications recorded for the period 1 January 2020 to 31 May 2021. At the time of writing (December 2021), this prediction seems plausible using a common-sense ‘test’, i.e., that the pandemic is still impacting globally on all aspects of society. In fact, it may also seem plausible that the lower bound predicted by the model is overly ‘pessimistic’ given the discovery on 25 November 2021 of a new COVID-19 variant, omicron (Squazzin, 2021), and the impact that this discovery has had on the daily lives of citizens around the globe. This is a reminder of the importance of uncertainty bounds in forecasting to the extent that unexpected events (e.g., the omicron variant) may occur within already unprecedented conditions (e.g., the COVID-19 pandemic), creating conditions which may alter expected outcomes. In the case of the omicron variant, a position of increasing scientific certainty about the behavior of the novel coronavirus is disrupted, and policymakers and the public turn to science with renewed interest and expectation. It would be instructive to reapply the testing of forecasting models, best-fit analysis and the application of the most suitably growth model to determine whether and how this re-emergence of the importance of science amidst the pandemic shapes the growth of scientific literature.

The analysis of forecasted growth by access (i.e., OA versus non-OA, and green versus gold OA), shows the following: (1) that the number of non-OA publications will keep pace with OA publication; and (2) that gold OA, after an initial lag in growth, shows stronger future growth than green OA over the forecast period. This may support findings that publishers invested in, and modified, processes to make non-OA publishing as attractive as OA in terms of the benefits for the scientific community and others (cf., Horbach, 2020, 2021). Findings that publishers have increased the speed of peer review and, by implication, decreased the time from submission to publication, support the notion that non-OA publishers have adapted. Temporarily lowered paywalls and access only to those articles that relate to COVID-19 (while other articles remain behind paywalls) are evidence of additional strategies and may account for the continued growth in non-OA publications.

That gold OA is predicted to grow at a much faster rate than green OA further suggests adaptations are being made by scholarly publishers to protect viable scholarly publishing models without ‘losing out’ to the demands and needs of scientists and attentive publics during the COVID-19 pandemic. However, caution is warranted based on findings that most preprints are eventually published as journal articles (Fraser et al., 2020). This category of preprints is not included in the green OA category in Dimensions because the allocation of publications to categories is mutually exclusive. Further research that investigates which non-OA publishers have responded, how they have adapted their models (temporarily or permanently), and which takes into account multiple versions of scientific articles, would be instructive in terms of assessing the degree to which the COVID-19 pandemic has disrupted the scholarly publishing system in any systemic manner.

The forecasts show growth across all the scientific fields analyzed. Not unexpectedly, the Medical & Health Sciences literature is dominant and is predicted to remain so. However, the forecasts also show relatively higher growth for the Education and for Studies on Human Society fields. Both fields relate to the study of social issues and phenomena. Their higher rate of growth may be explained by the increasing attention being paid to the societal impact of the COVID-19 pandemic, while other issues such as the economic and health impact obviously remain on the radar. Again, it would be instructive to see how the omicron variant and the panic it sparked in some parts of the world impact on the focus of science by disciplinary field. One could expect an increase in the Medical & Health Sciences field as the attention of the scientific community turns towards understanding the effects of the mutated virus with regard to its transmissibility, severity of infection, immunity evasion, and the like. At the same time, concerns in other parts of the world, particularly in South Africa, are focused on the economic and social impact of the travel restrictions imposed on the region, particularly the impact on jobs and economic recovery in a country heavily dependent on tourism from northern countries during the summer season.

This paper includes various analyses of the employed forecasting methods. The fitting performance evaluations were complemented by the cross-validation analyses to ensure that the proposed models performed well in forecasting. The models were tailored to each specific datasets and differences in performance show that considering a different model for each setting is preferable to a one-size-fits-all approach.

## Conclusions

In conclusion, we return to our research questions. First, we asked whether growth in COVID-19 science during the pandemic followed the same patterns as the growth in scientific literature during ‘normal’ times. Both historical and forecast COVID-19 data show that the growth patterns are different from ‘normal’ times, with a more rapid growth. As noted in the introduction, the average growth in scientific publications is estimated to double approximately every 17 years (Bornmann et al., 2021). In contrast, the COVID-19 literature growth is expected to double during the next 10 months. Moreover, the average growth in scientific publications was estimated at 4% per annum (Bornmann et al., 2021). The data at hand pointed towards a growth of 846% from May 31st 2020 until May 31st 2021, which again shows the discrepant growth of COVID-19 scientific output when compared to ‘normal’ times.

We are interested in any observable disruptions to the scholarly communication system during the COVID-19 pandemic. Our results show an observable quantum and rate of growth in open access publishing during the pandemic, but that this is matched in both respects by non-open access scholarly publishing. Furthermore, there is no sign that green open access articles are gaining greater momentum compared with gold open access journal articles during the COVID-19 pandemic. The answer to both open access questions is therefore negative—we did not observe a shift towards (green) open access during the pandemic. In terms of academic fields, there is an increase in interest by the scientific community in the social dimension of the pandemic, but not noticeably at the expense of publications in other scientific fields. How will these differences evolve over time will depend on developing a deeper understanding of the attention of both scientists and attentive publics shift as the pandemic progresses, as well as how scholarly publishers continue to respond the expectations of scientists under pressure to provide new knowledge and, as a consequence, greater certainty about the impacts and duration of the pandemic. These insights provide tentative answers to the research questions regarding differences in scientific fields, which extend an invitation to expand the current analysis.

## Supplementary Information

Below is the link to the electronic supplementary material.

Supplementary file1 (DOCX 445 kb)

## Footnotes

1 After a random inspection of records tagged as books and monographs, we observe that records tagged as monographs are single-authored books, while those tagged as books are group-authored or edited books.
