# Supplementary material for: COVID-19 and the scientific publishing system: growth, open access and scientific fields
Source: Scientometrics. 2022 Oct 10;128(1):345–62. doi: 10.1007/s11192-022-04536-x (PMC9548429; doi:10.1007/s11192-022-04536-x)
Supplement: Supplementary file 1 — Supplementary file1 (DOCX 445 kb) [file 11192_2022_4536_MOESM1_ESM.docx]

Supplementary material: COVID-19 and the scientific publishing system: Growth, open access and scientific fields

**Gabriela F. Nane^[[1]](#footnote-1)^, Nicolas Robinson-Garcia^2^, François van Schalwyk^3^, Daniel Torres-Salinas^2^**

^1^ Delft Institute of Applied Mathematics (DIAM), TU Delft, Delft, Netherlands

^2^ EC3 Research Group, Information and Communication Studies department, University of Granada, Granada, Spain

^3^ DST-NRF Centre of Excellence in Scientometrics and Science, Technology and Innovation Policy, Centre for Research on Evaluation, Science and Technology, Stellenbosch University, Stellenbosch, South Africa

Contents

[Summary 1](#_Toc88557354)

[1. Growth models 2](#_Toc88557355)

[Figure S1. Fit of the exponential (A) and logistic growth models (B) to publications related to COVID-19 2](#_Toc88557356)

[Figure S2. Fit of the exponential and logistic growth models to Open Access publications (A) and non-Open Access publications (B) related to COVID-19 2](#_Toc88557357)

[Figure S3. Fit of the exponential and logistic growth models to Gold Open Access (A) and Green (only) Open Access publications (B) related to COVID-19 3](#_Toc88557358)

[2. Validation of models 4](#_Toc88557359)

[Figure S4. Validation for ARIMA and Exponential Smoothing models for all COVID-19 publications. 4](#_Toc88557360)

[Figure S5. Validation for ARIMA and Exponential Smoothing models for Open Access, non-Open Access, Gold and Green (only) COVID-19 publications. 4](#_Toc88557361)

[Figure S6. Validation for ARIMA and Exponential Smoothing models per scientific field. Field classification based on the Australian and New Zealand Standard Research Classification. It includes the 22 Fields of Research (FoR). 5](#_Toc88557362)

# Summary

Supplementary figures to the study ‘COVID-19 and the scientific publishing system: Growth, open access and scientific fields’ which aims at forecast the growth of COVID-19 literature to better understand the magnitude of the phenomenon and to what extent it has disrupted the scientific publishing system. Here we include figures related to the fitness of the most common growth models applied in scientometric literature analyzing the growth of publications, and the validation of the ARIMA and Exponential Smoothing models for the different times series analyzed: all COVID-19 literature, Open Access (OA) and non-OA, Gold OA and Green (only) OA, and by scientific fields.

# 1. Growth models

## Figure S1. Fit of the exponential (A) and logistic growth models (B) to publications related to COVID-19

| A | B |
| --- | --- |
| 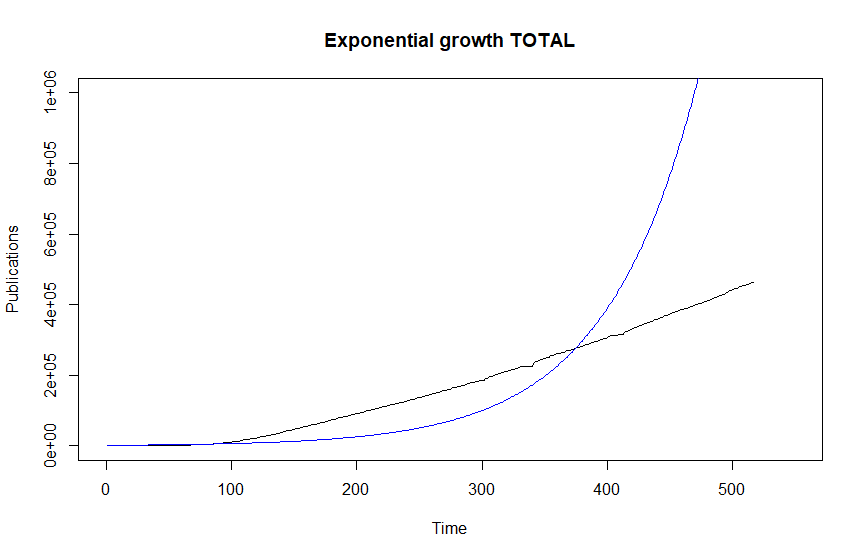 | 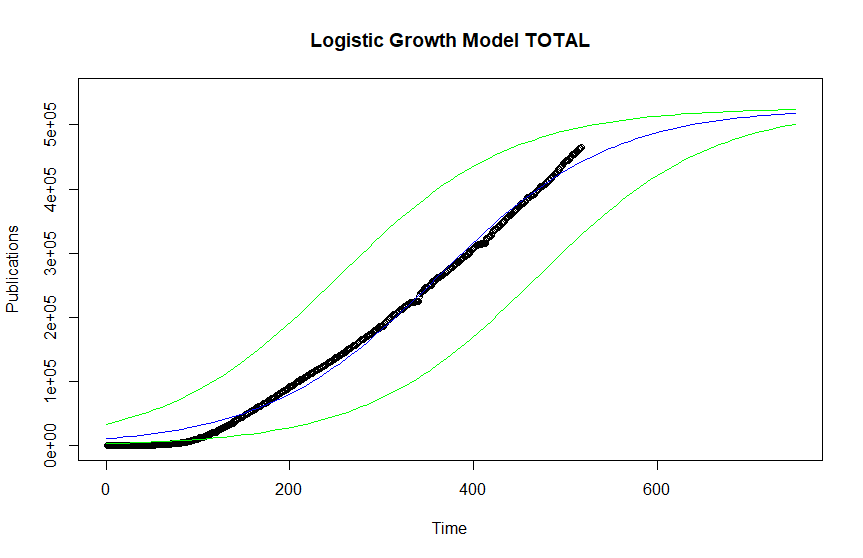 |

## Figure S2. Fit of the exponential and logistic growth models to Open Access publications (A) and non-Open Access publications (B) related to COVID-19

| A |  |
| --- | --- |
| 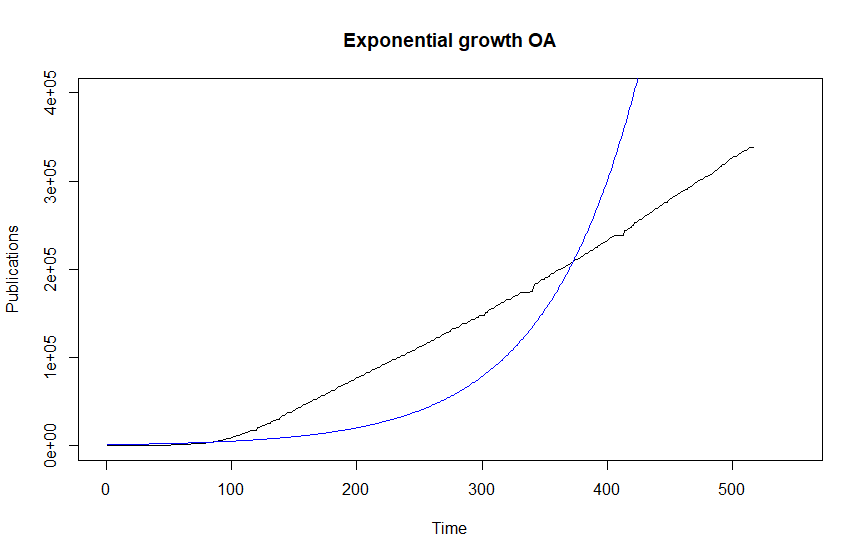 | 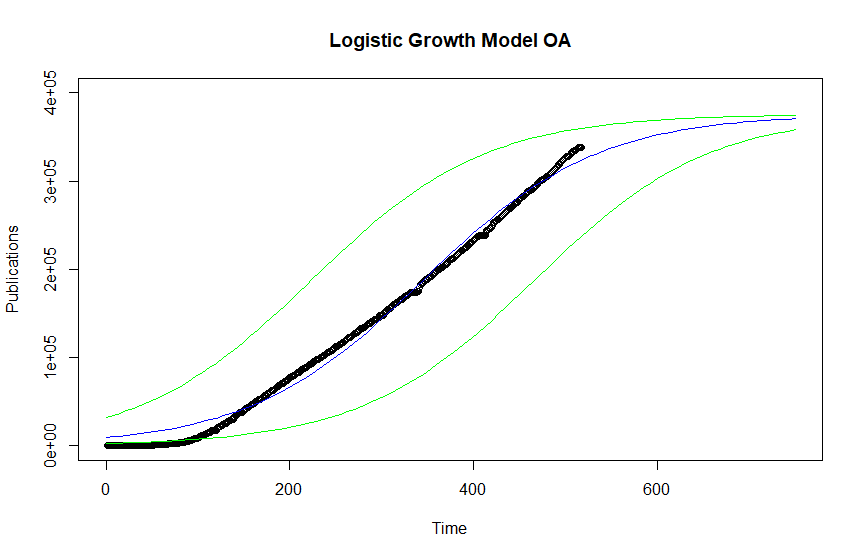 |
| B |  |
| 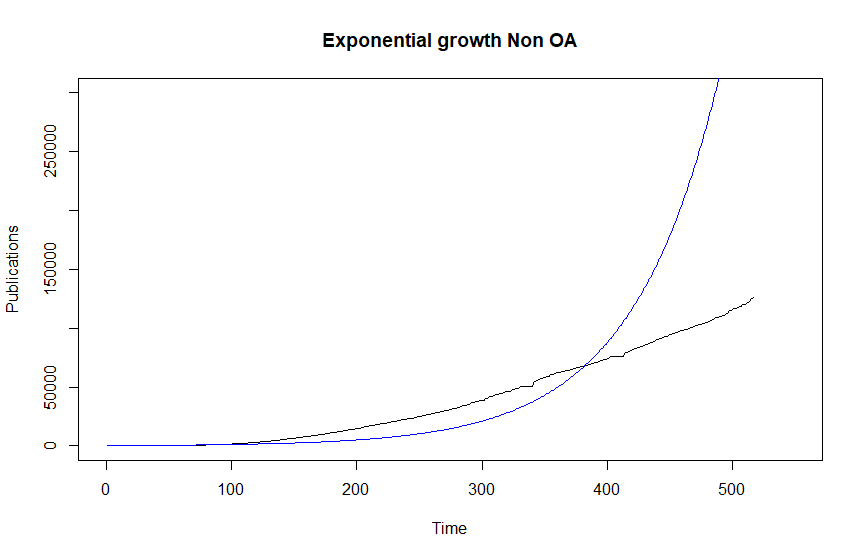 | 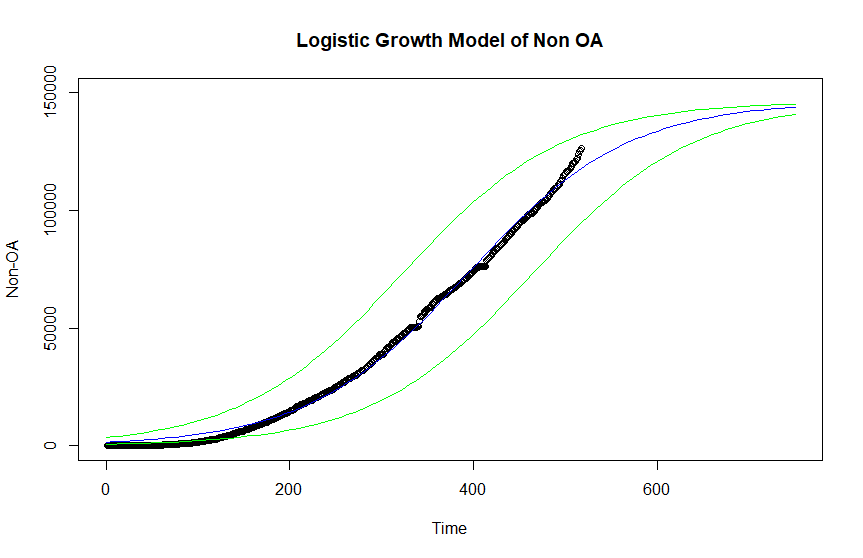 |

## Figure S3. Fit of the exponential and logistic growth models to Gold Open Access (A) and Green (only) Open Access publications (B) related to COVID-19

| A |  |
| --- | --- |
| 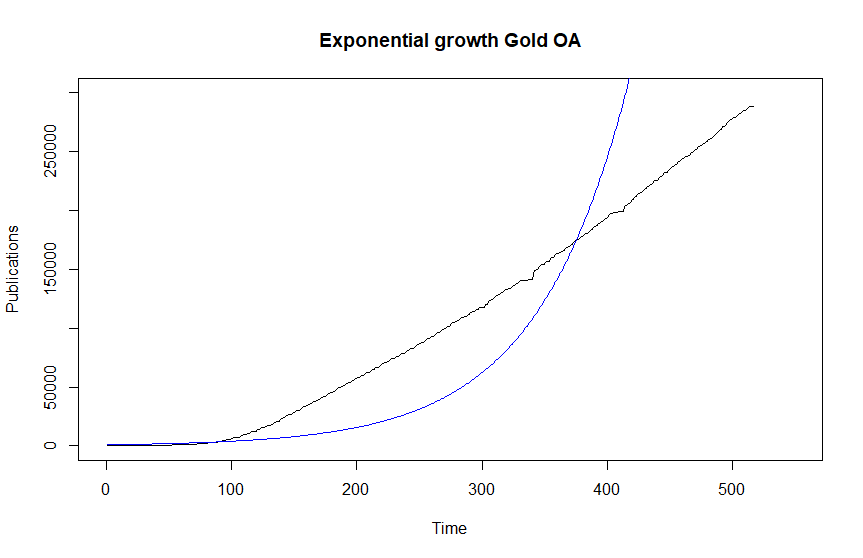 | 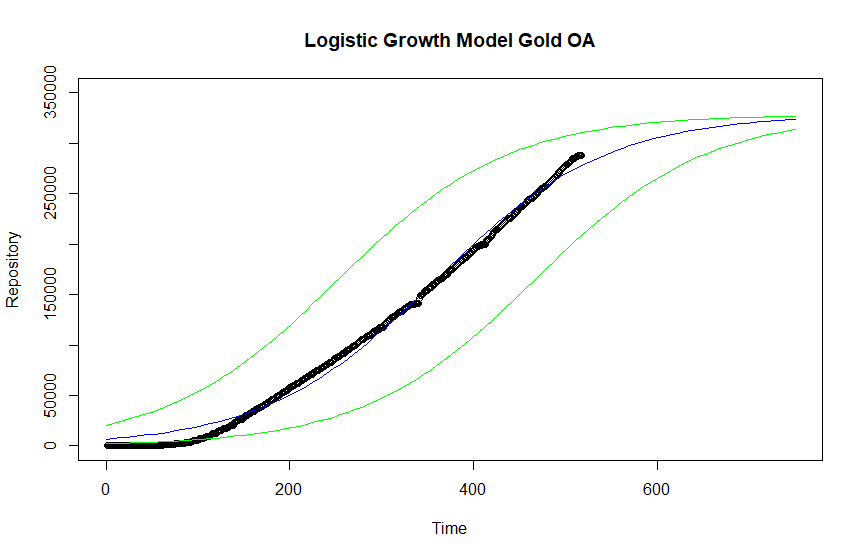 |
| B |  |
| 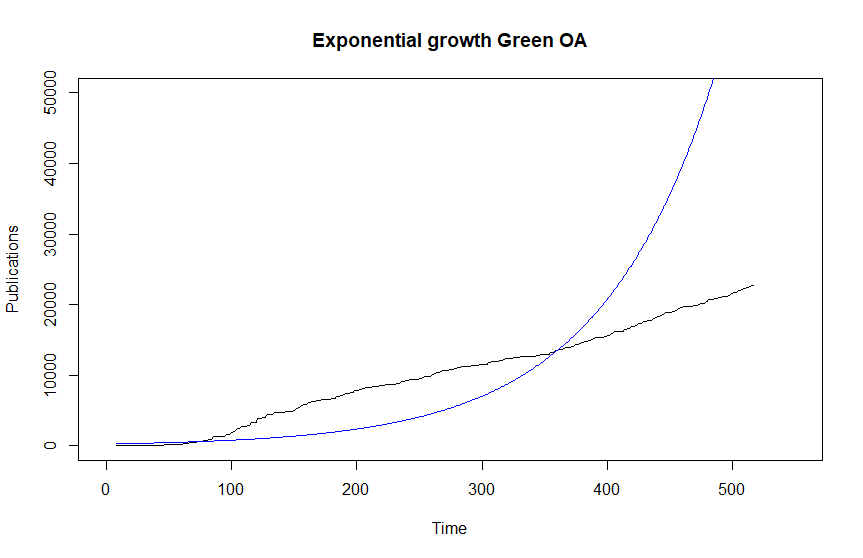 | 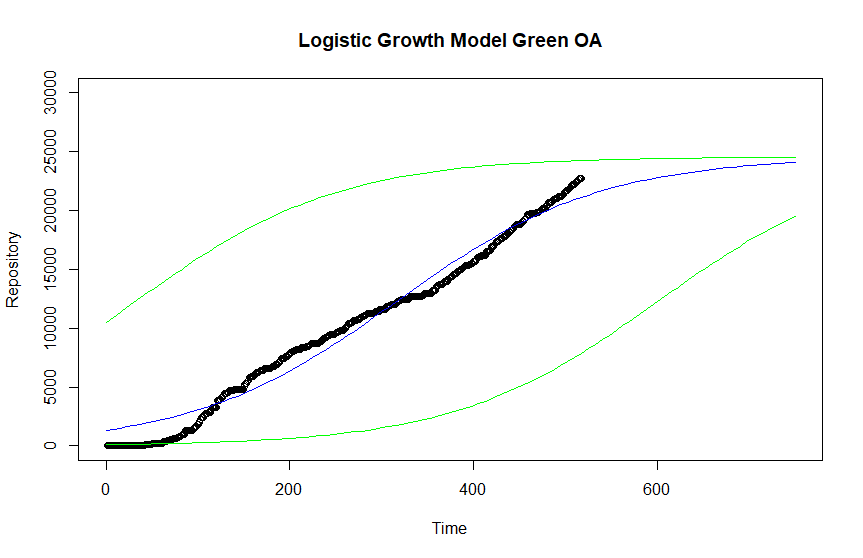 |

# 2. Validation of models

## Figure S4. Validation for ARIMA and Exponential Smoothing models for all COVID-19 publications.

| 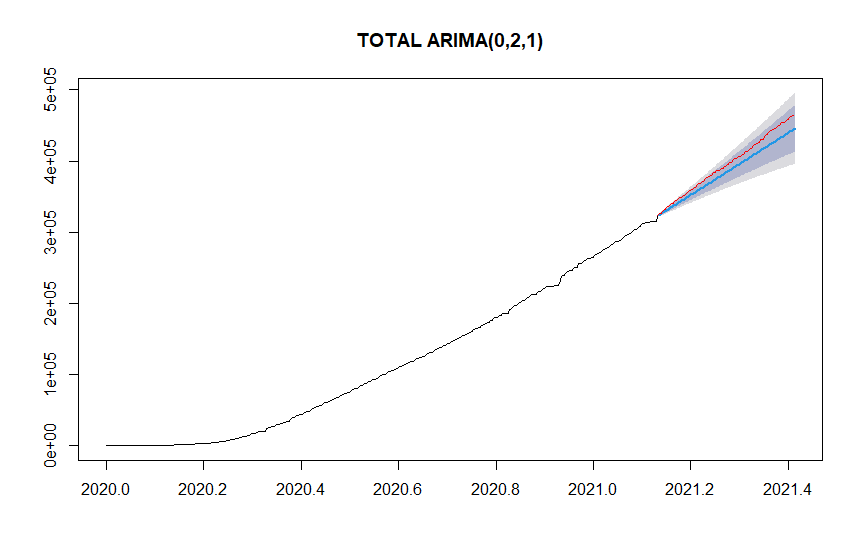 | 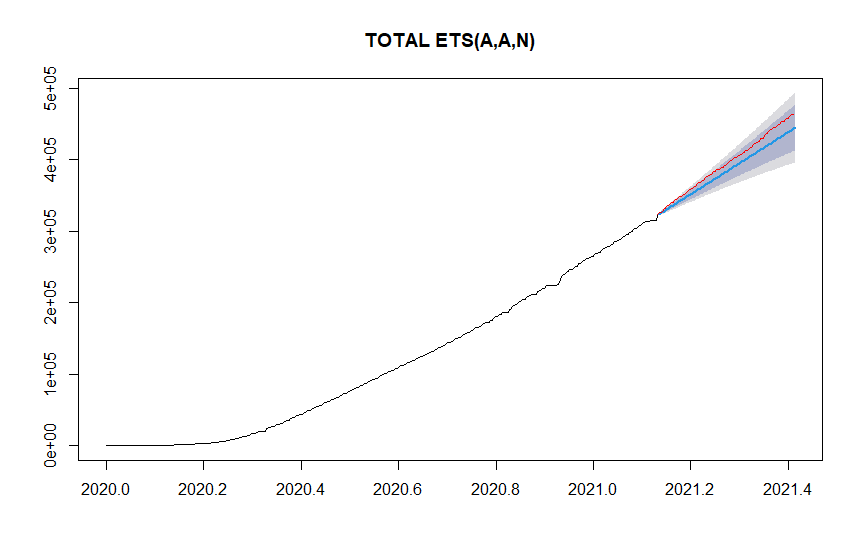 |
| --- | --- |

## Figure S5. Validation for ARIMA and Exponential Smoothing models for Open Access, non-Open Access, Gold and Green (only) COVID-19 publications.

| 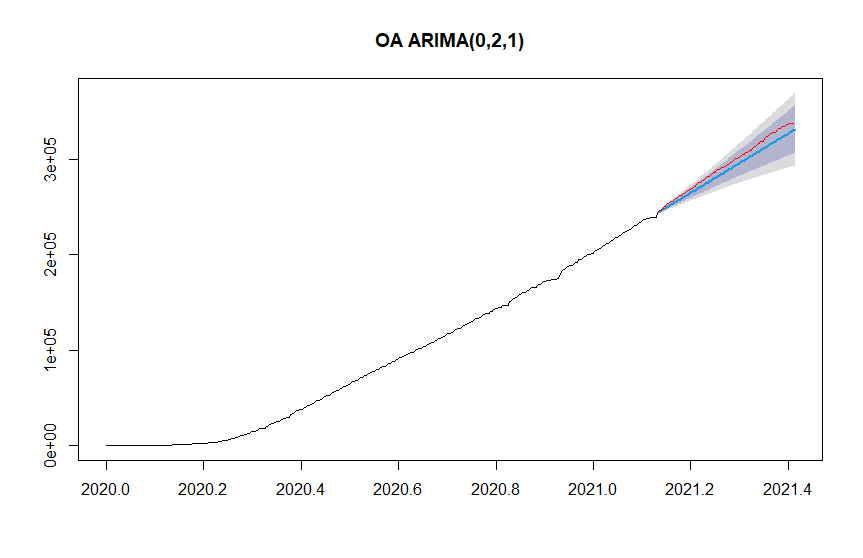 | 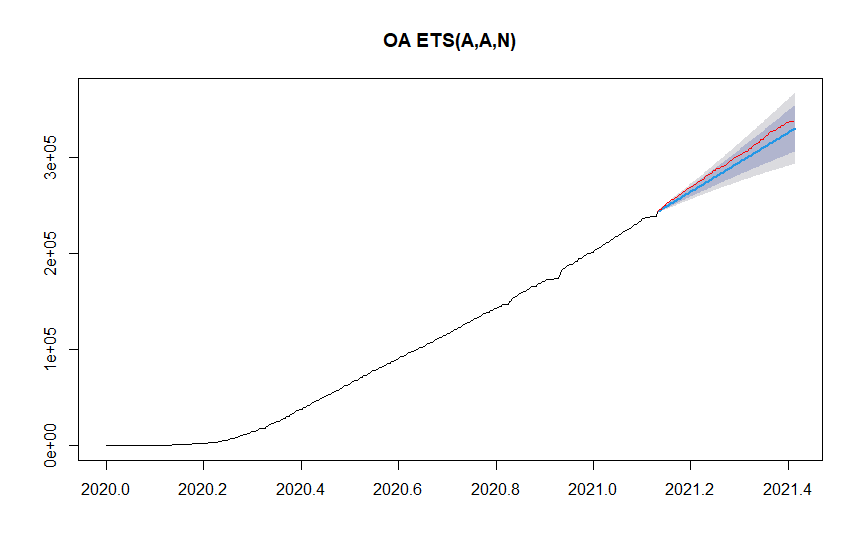 |
| --- | --- |
| 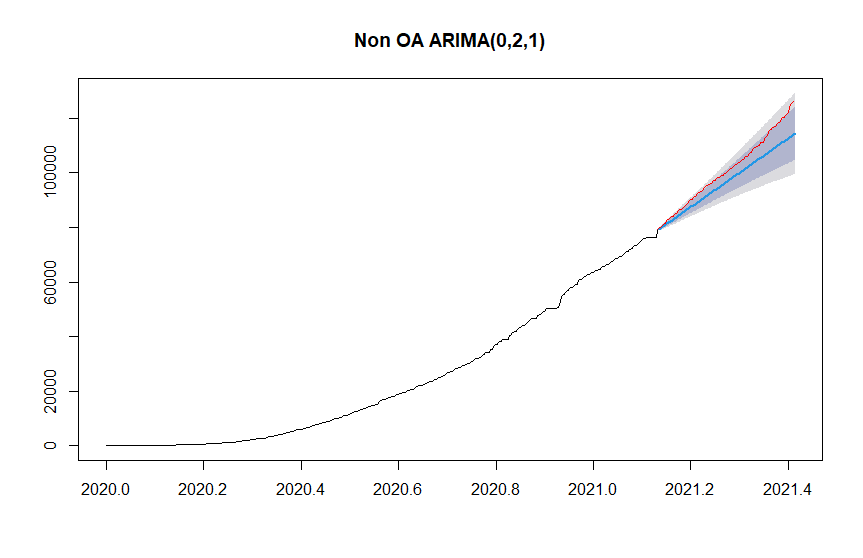 | 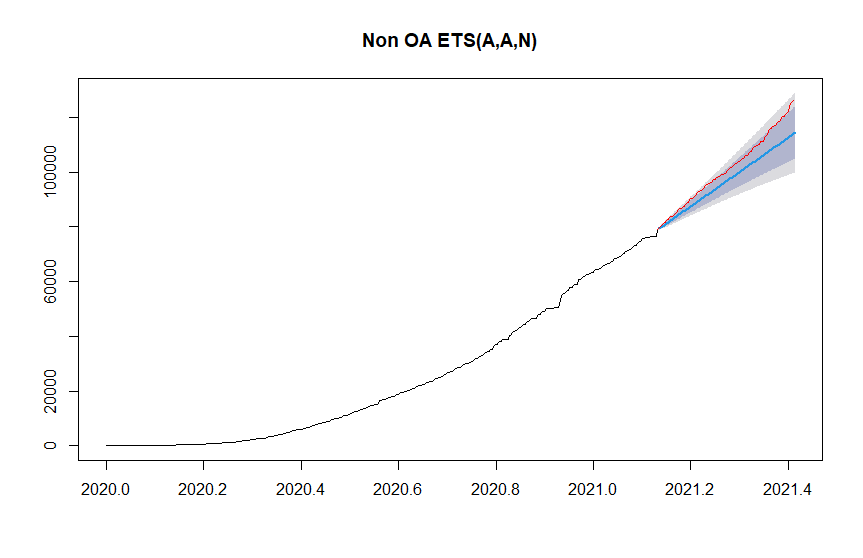 |
| 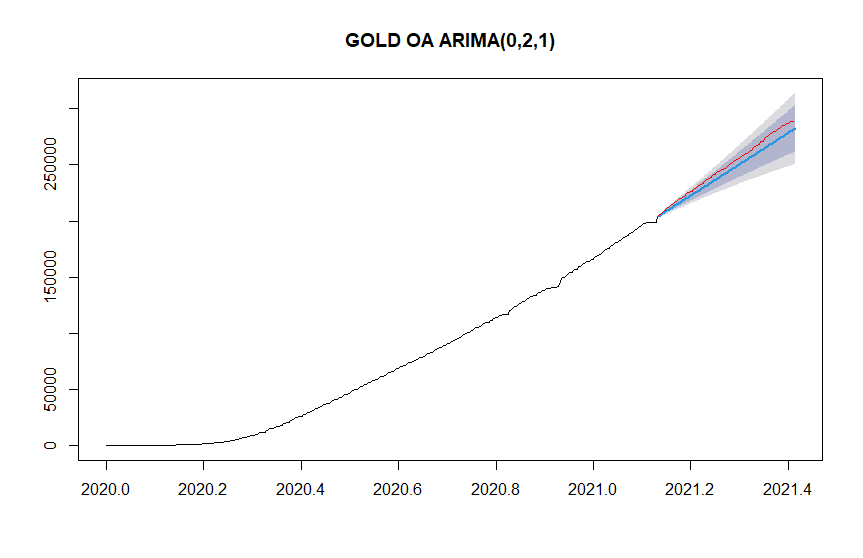 | 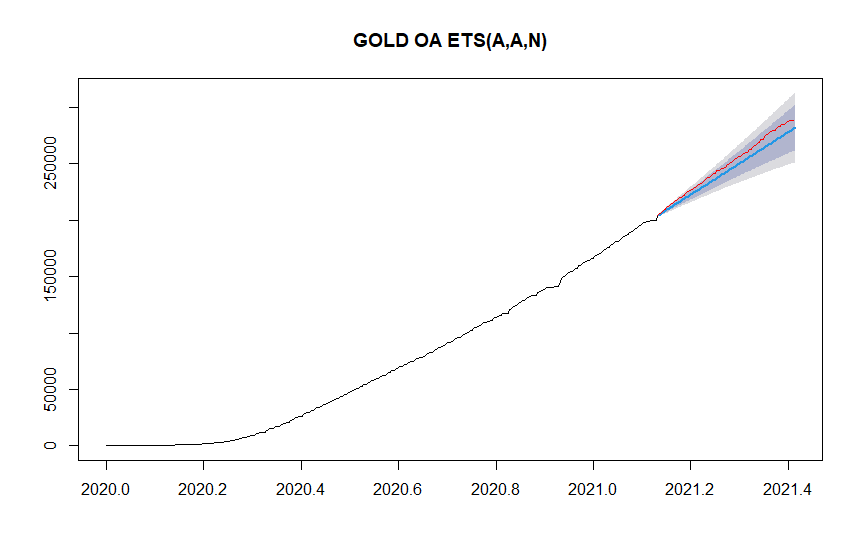 |
| 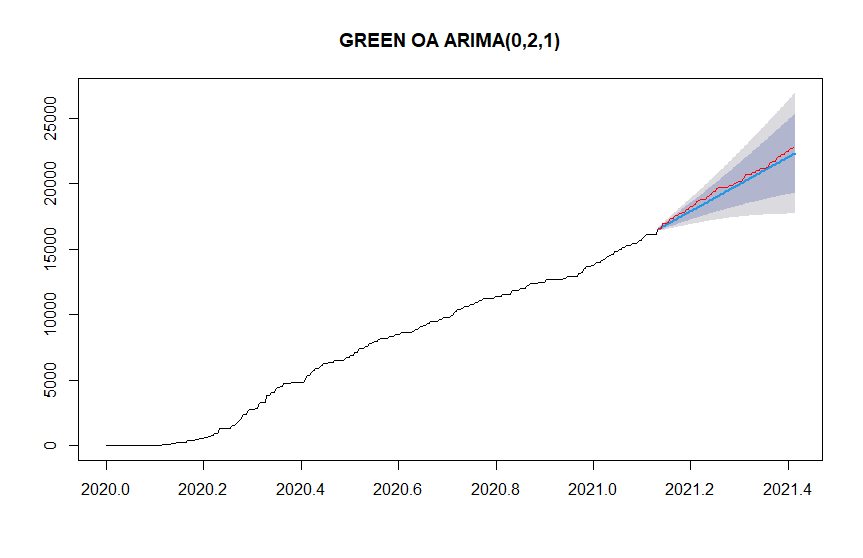 | 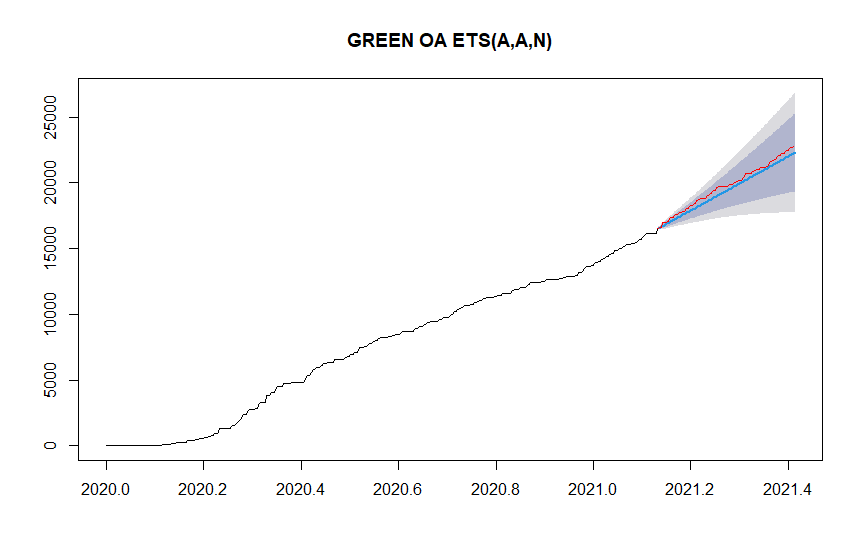 |

## Figure S6. Validation for ARIMA and Exponential Smoothing models per scientific field. Field classification based on the Australian and New Zealand Standard Research Classification. It includes the 22 Fields of Research (FoR).

| 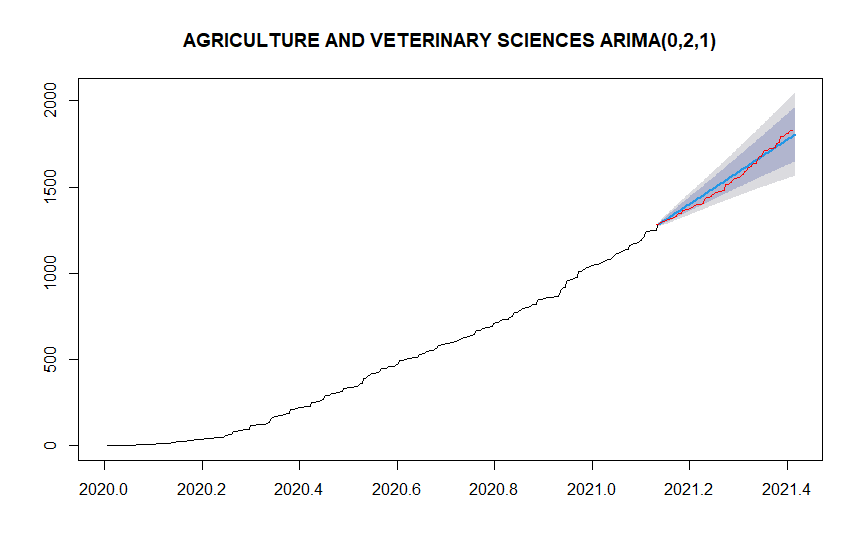 | 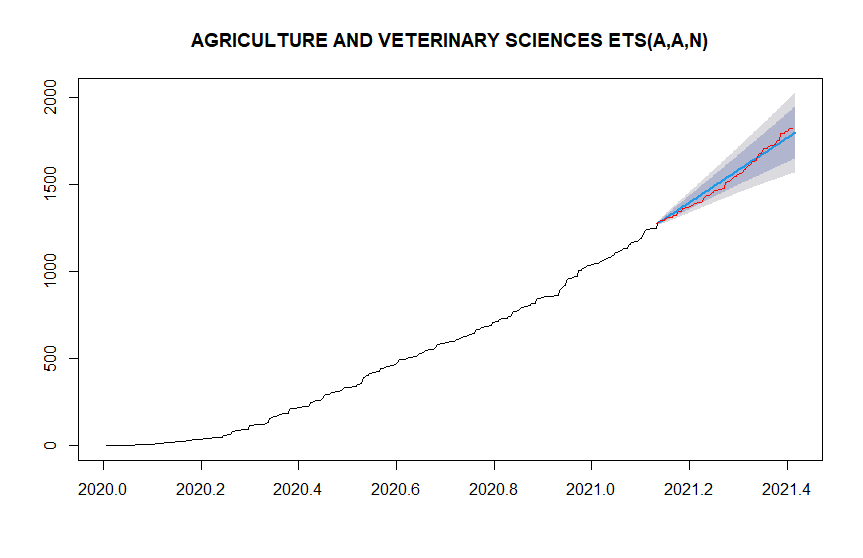 |
| --- | --- |
| 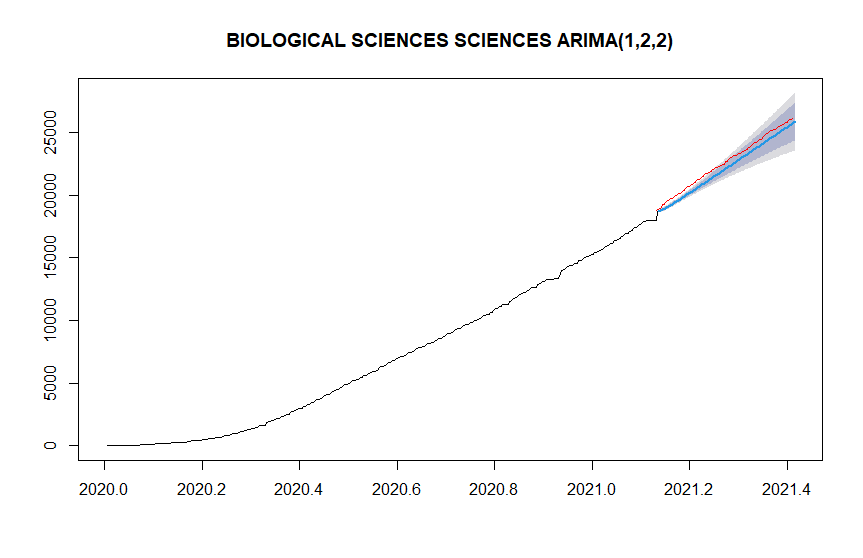 | 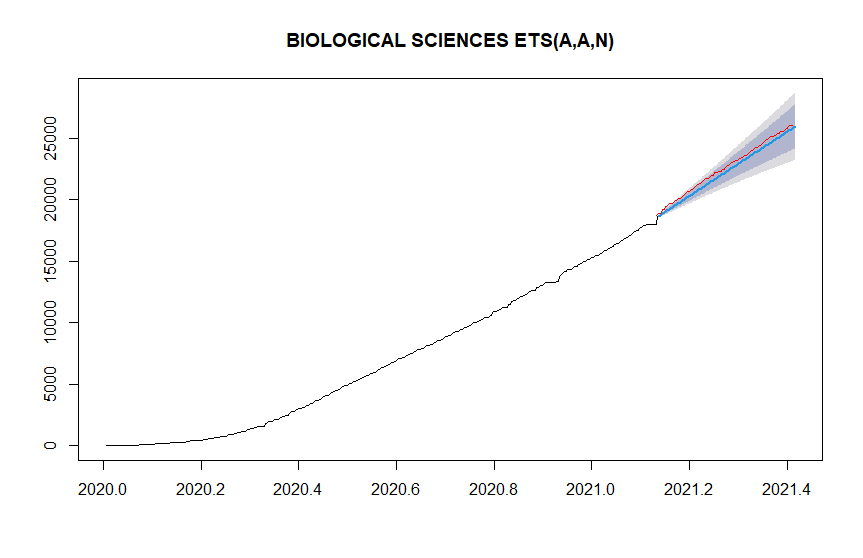 |
| 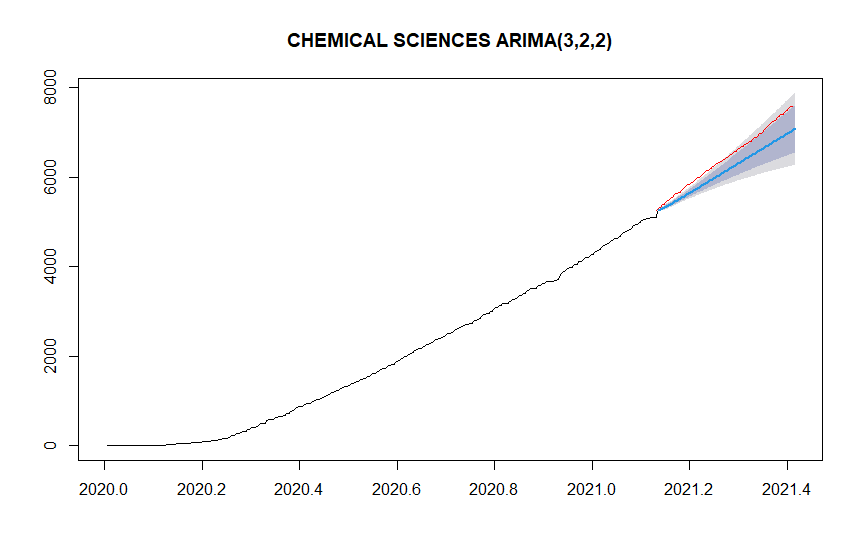 | 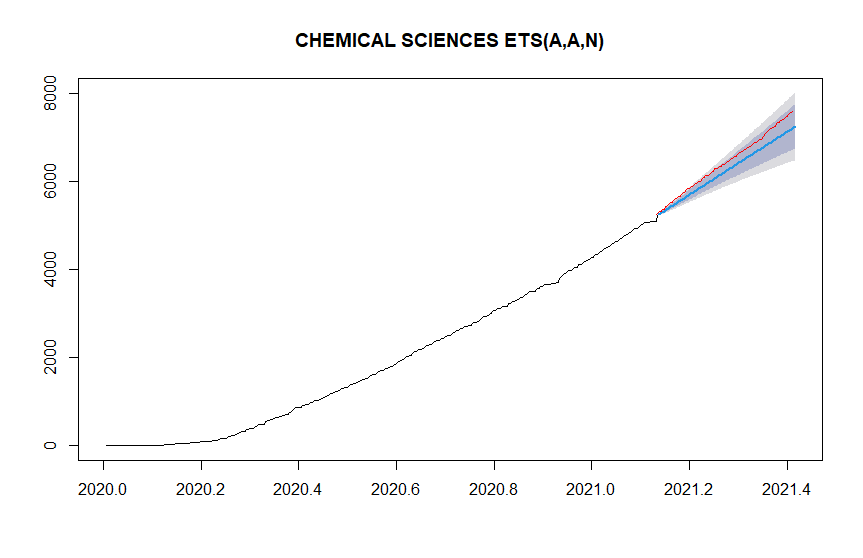 |
| 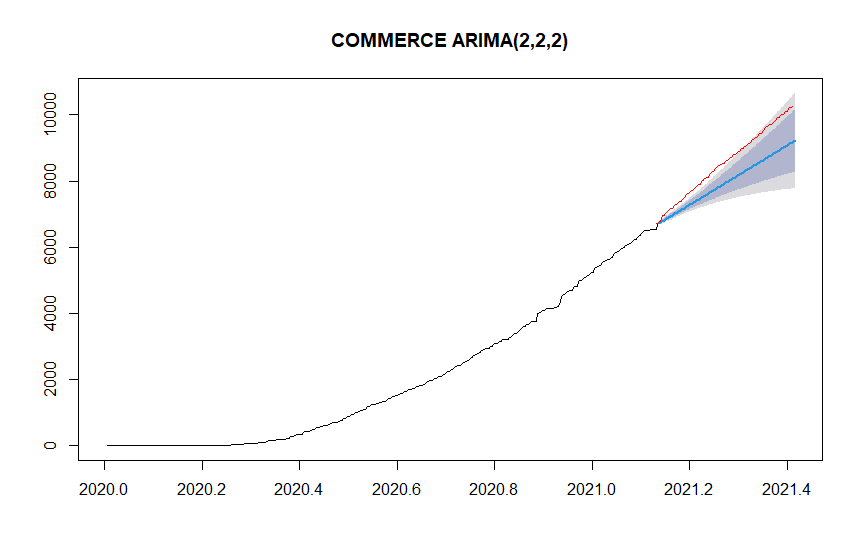 | 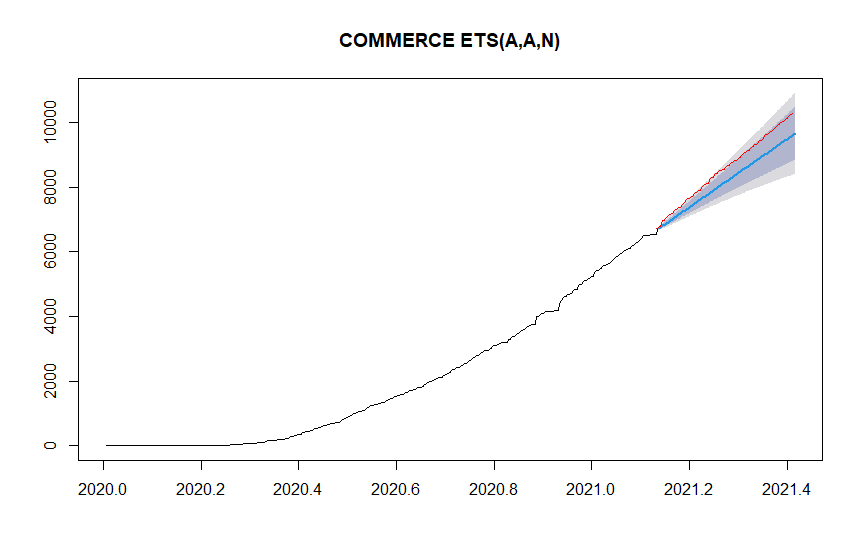 |
| 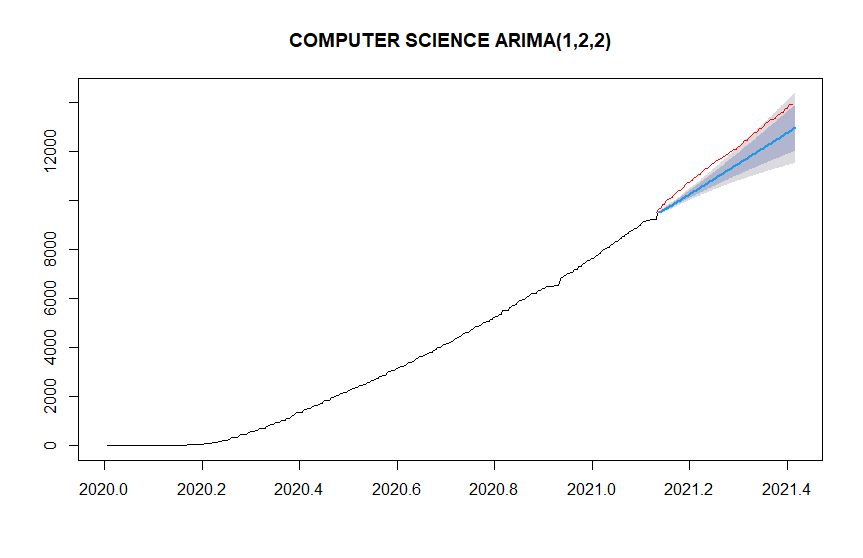 | 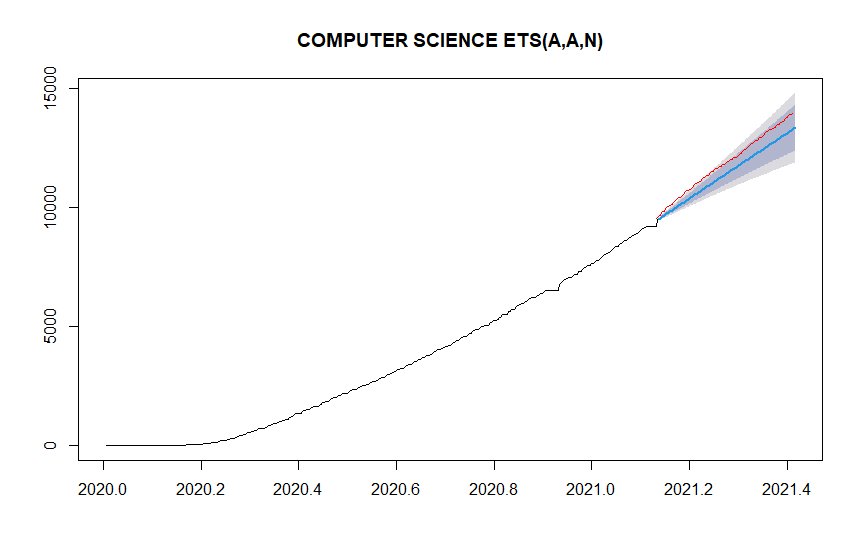 |
| 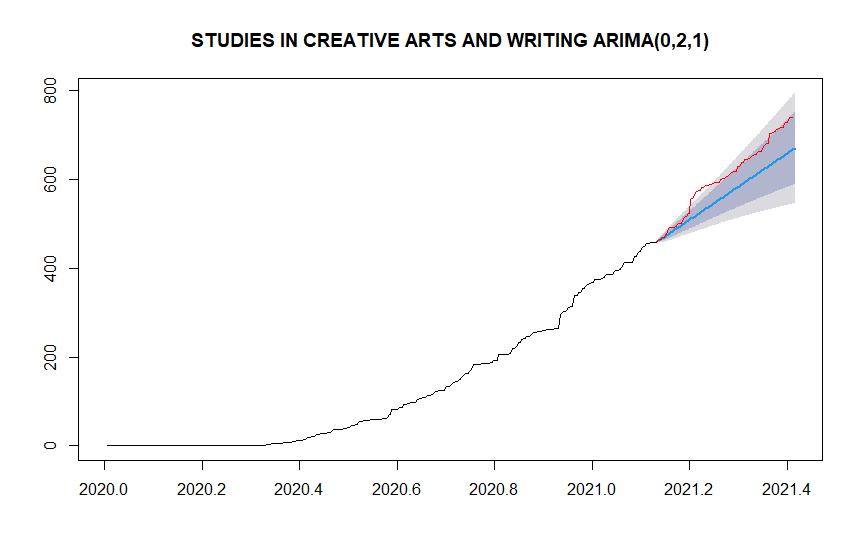 | 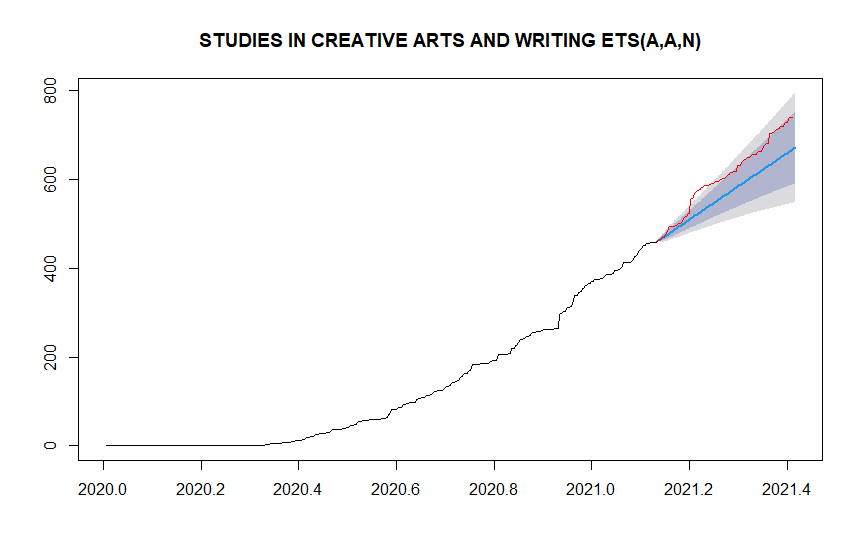 |
| 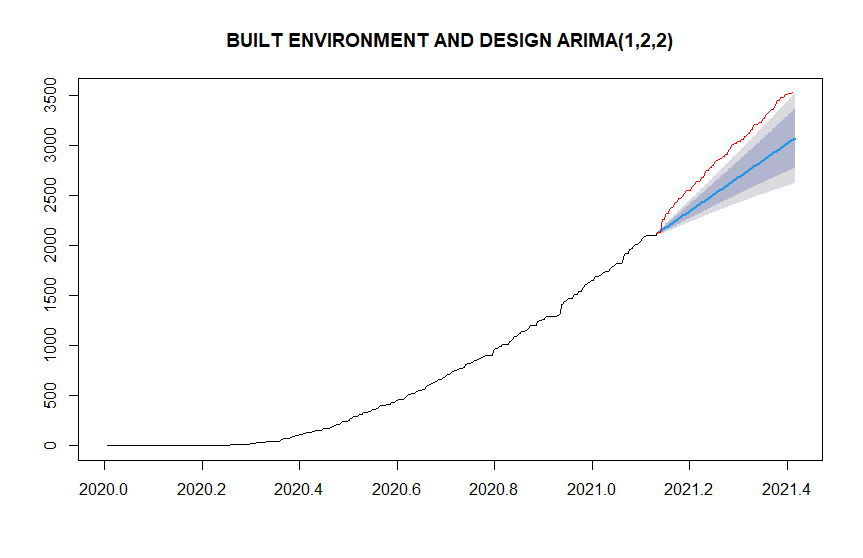 | 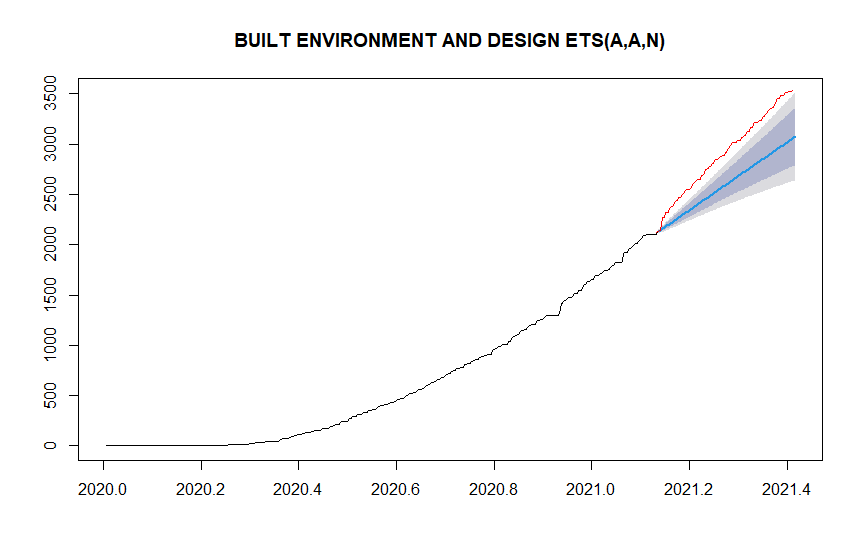 |
| 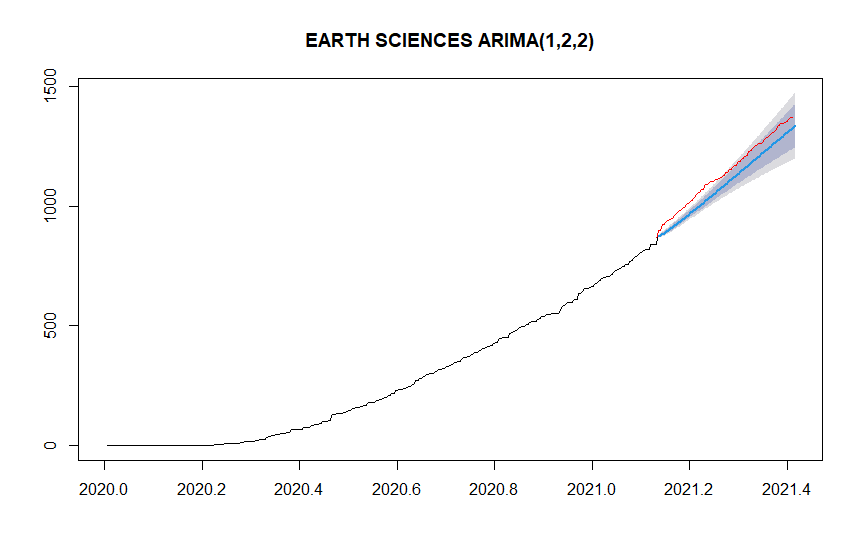 | 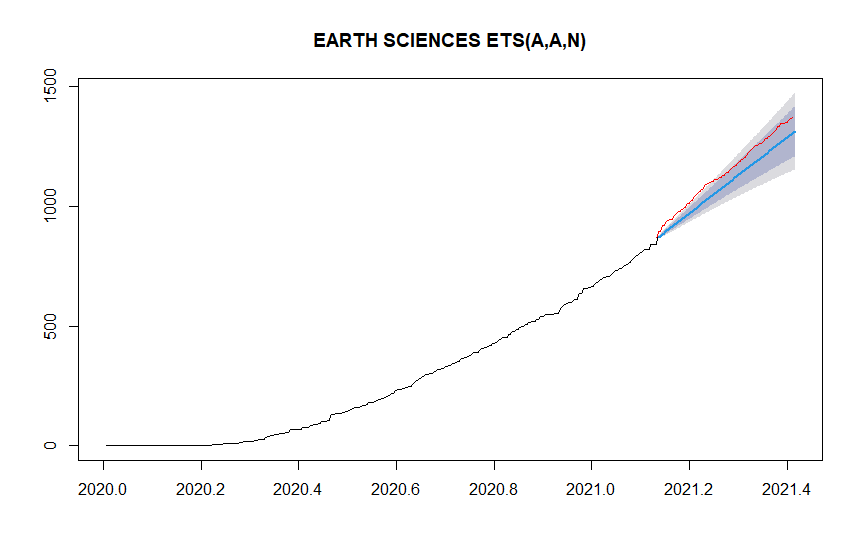 |
| 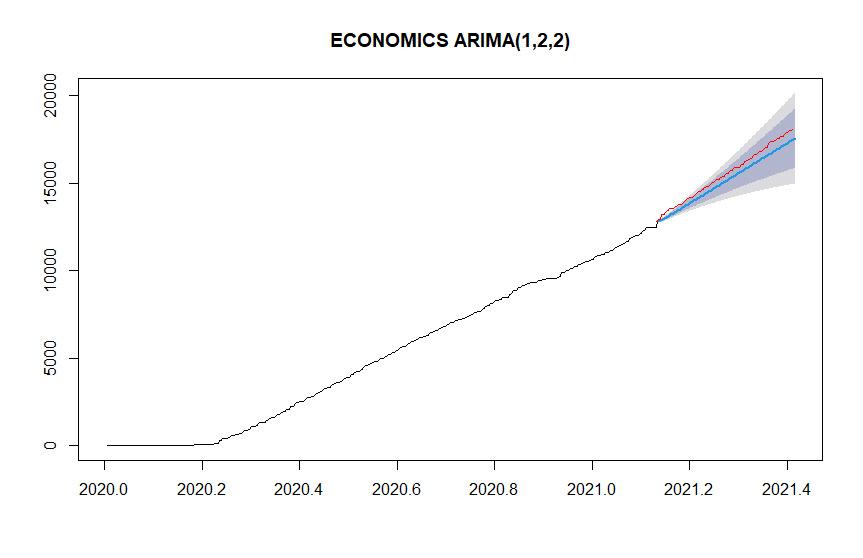 | 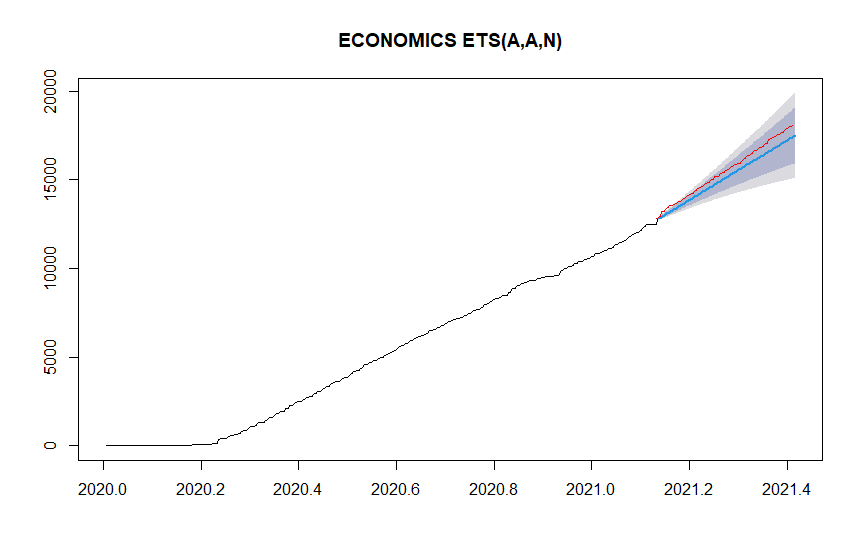 |
| 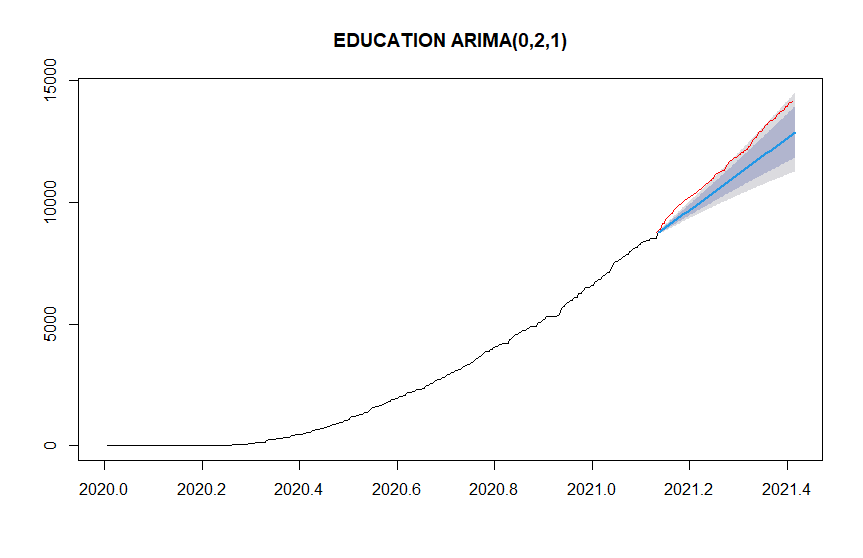 | 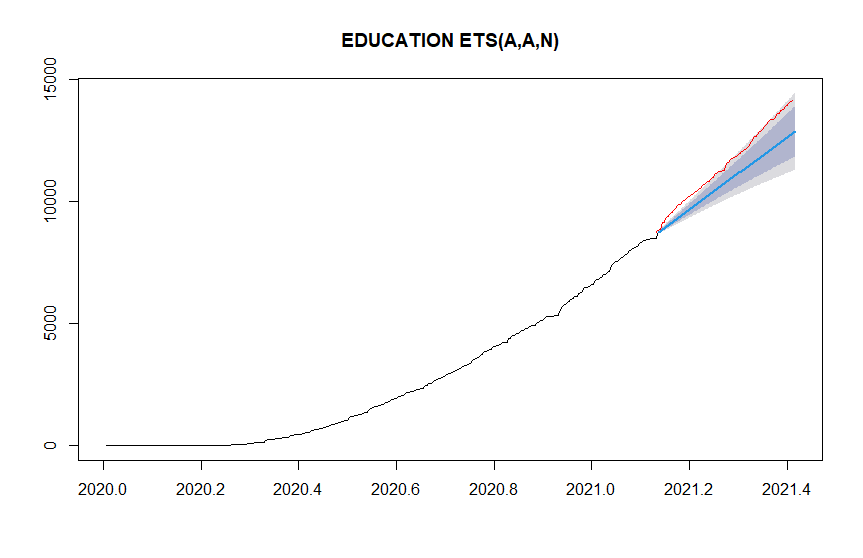 |
| 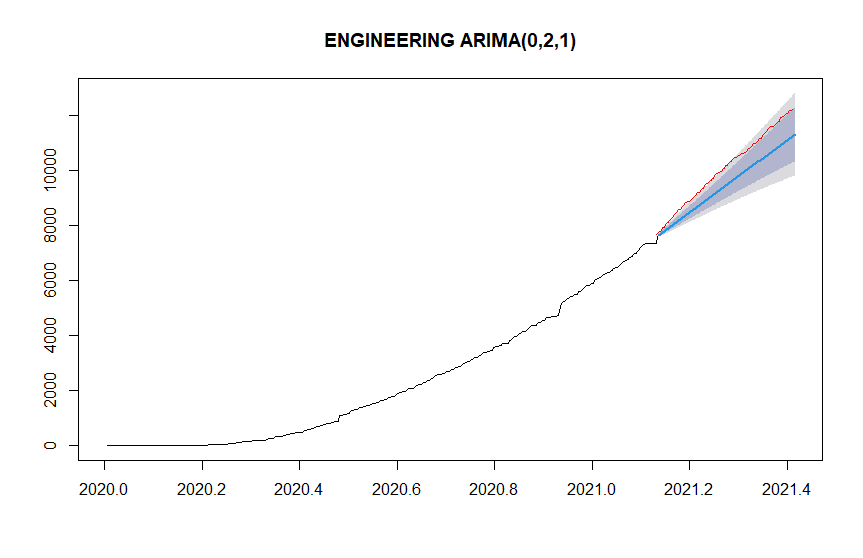 | 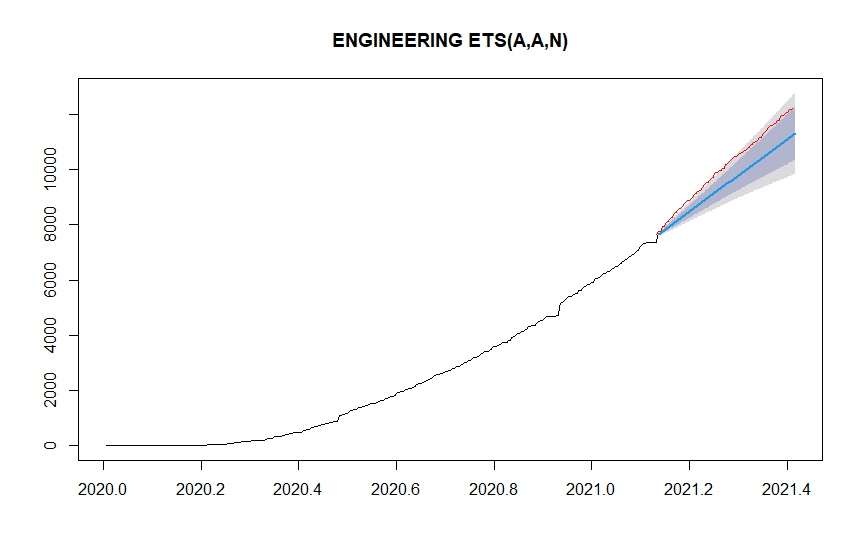 |
| 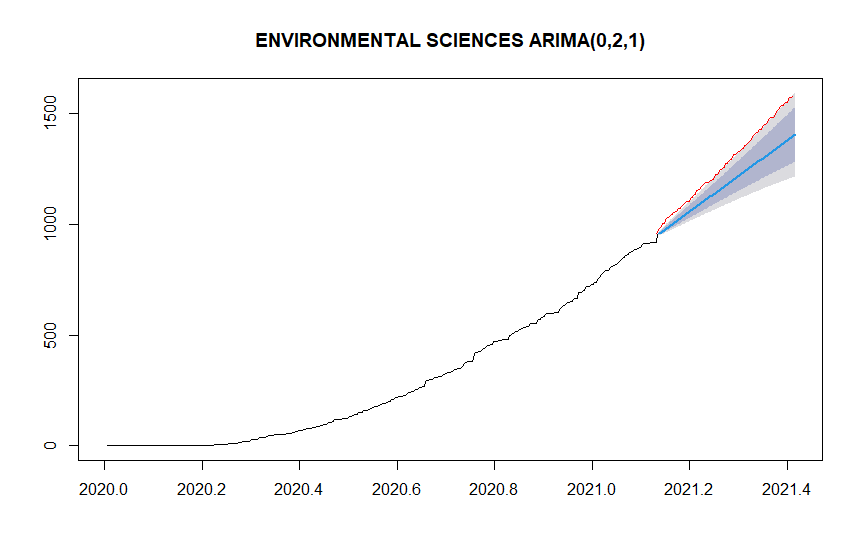 | 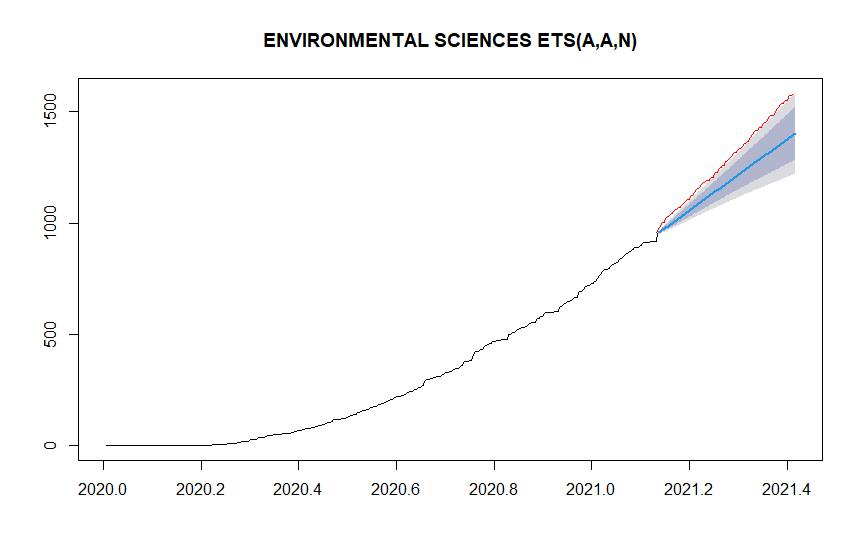 |
| 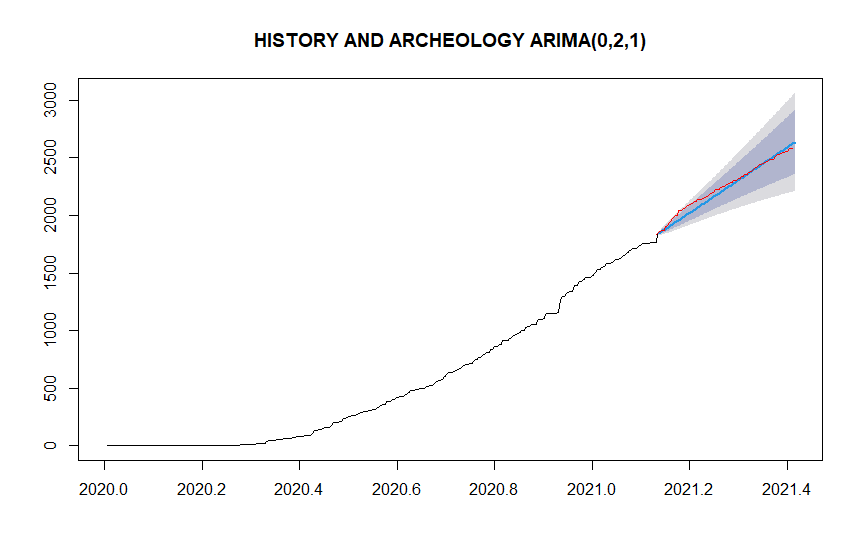 | 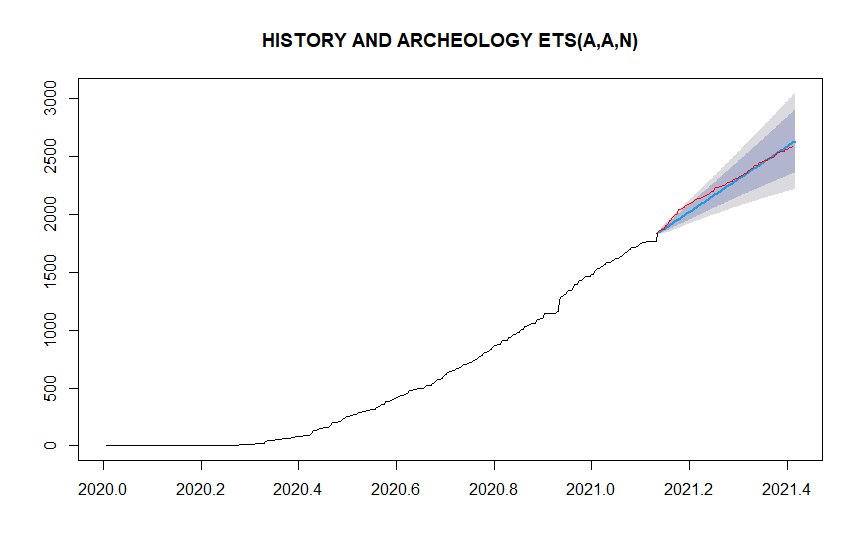 |
| 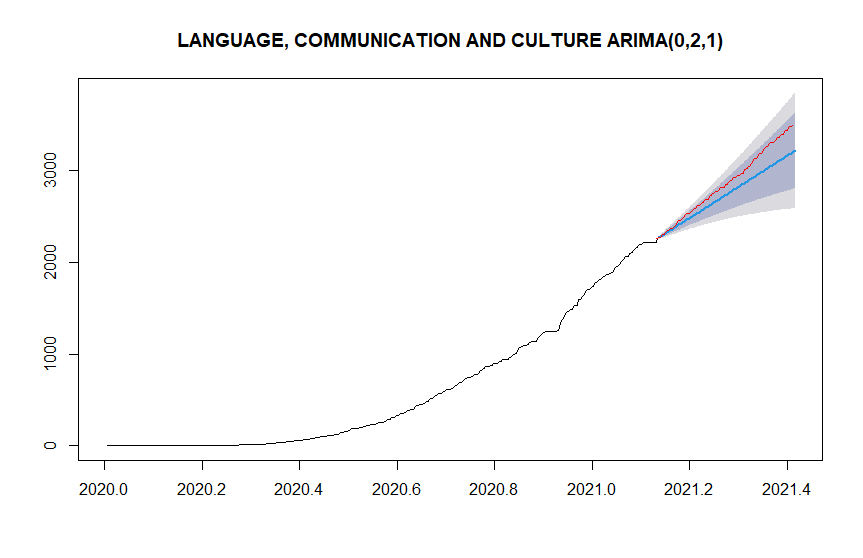 | 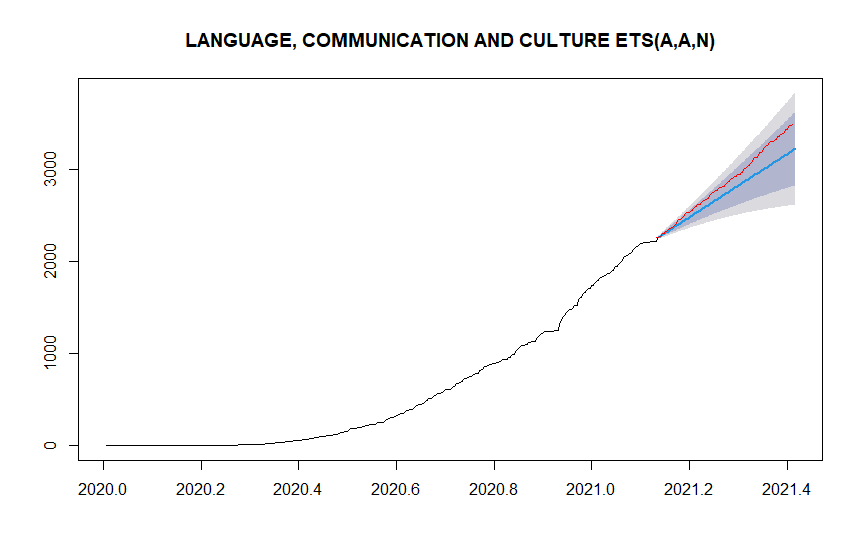 |
| 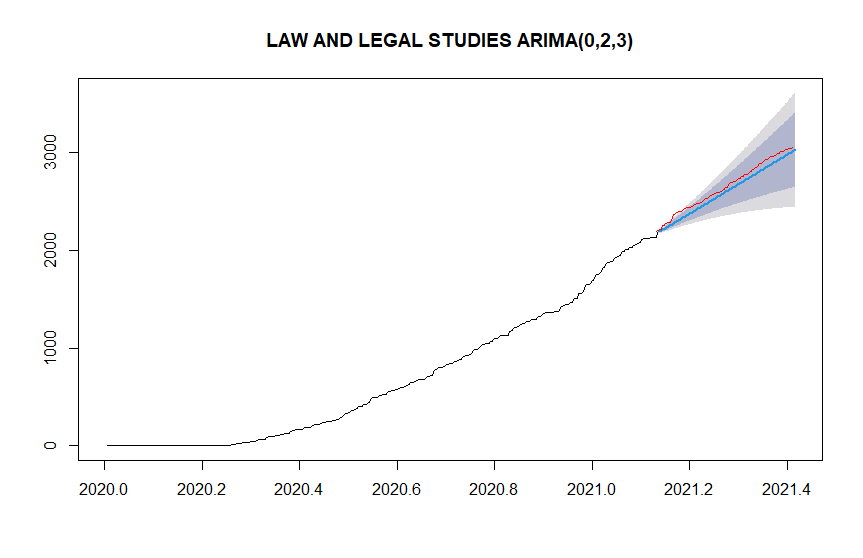 | 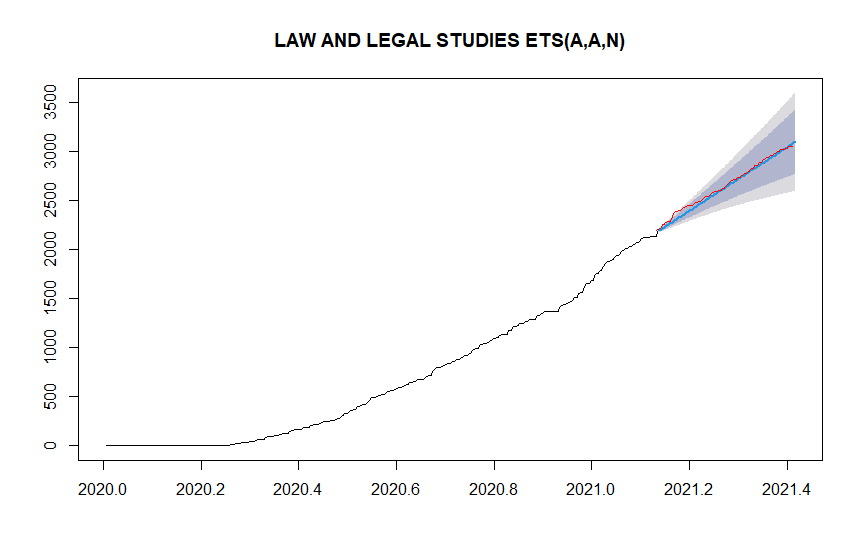 |
| 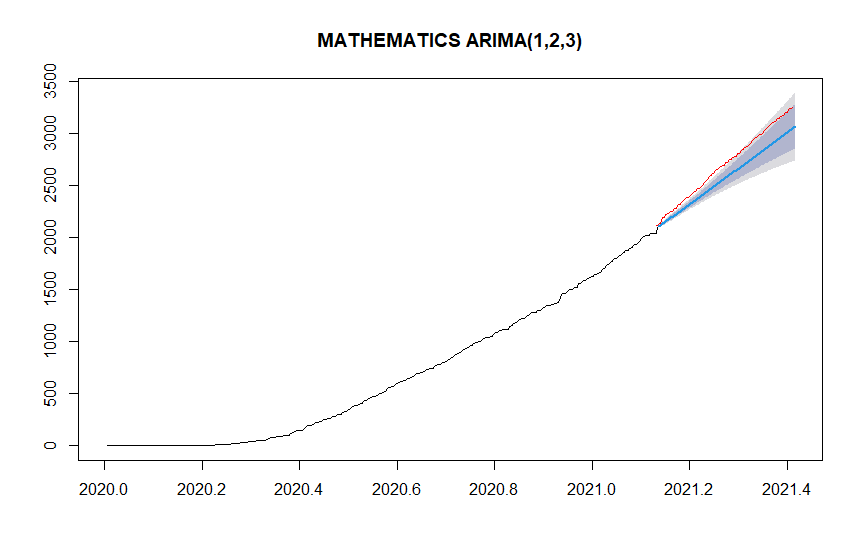 | 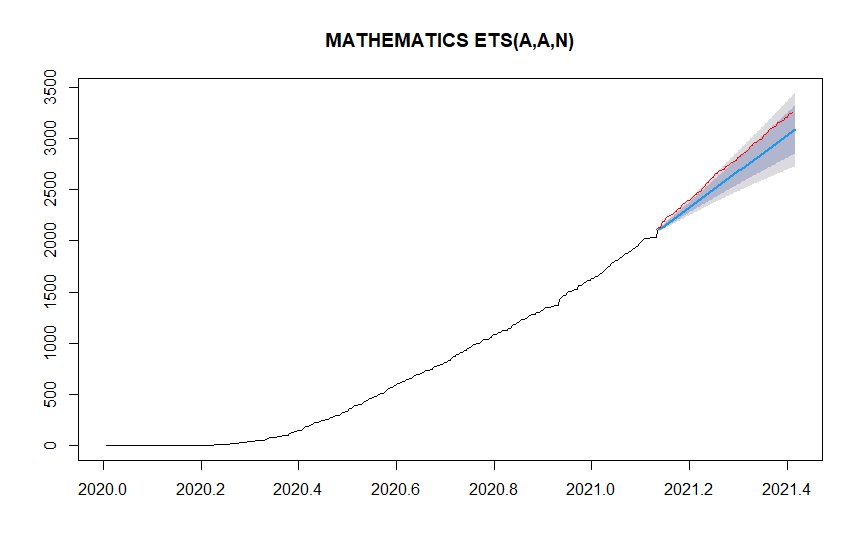 |
| 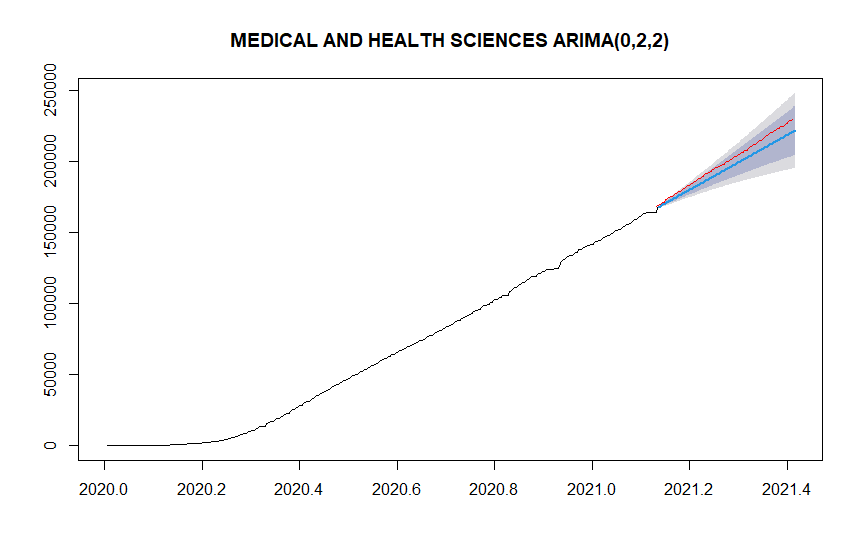 | 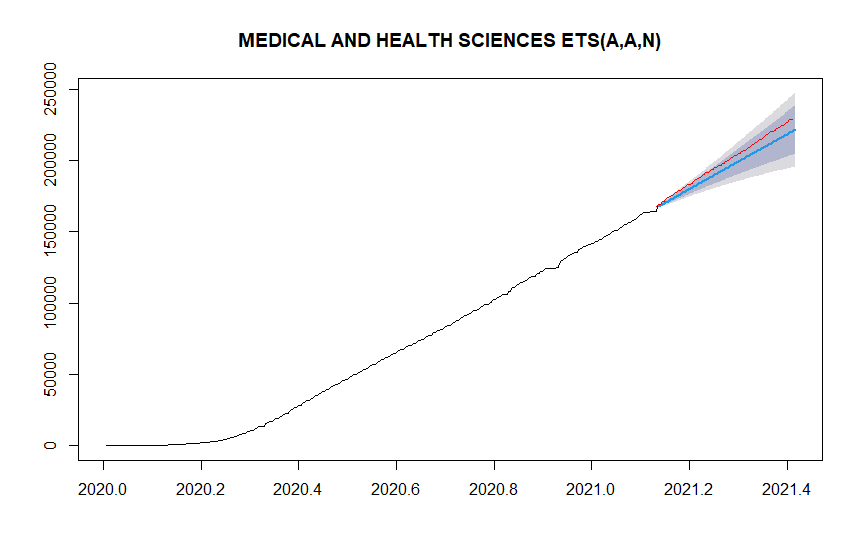 |
| 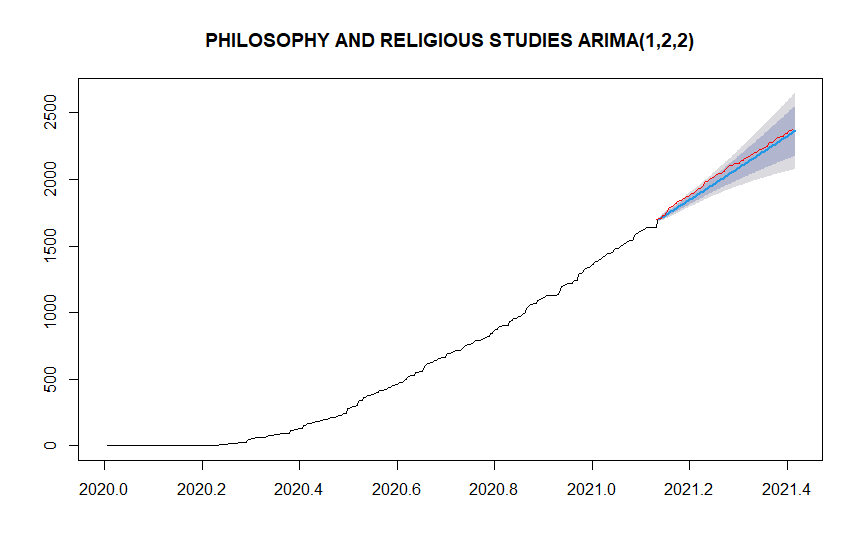 | 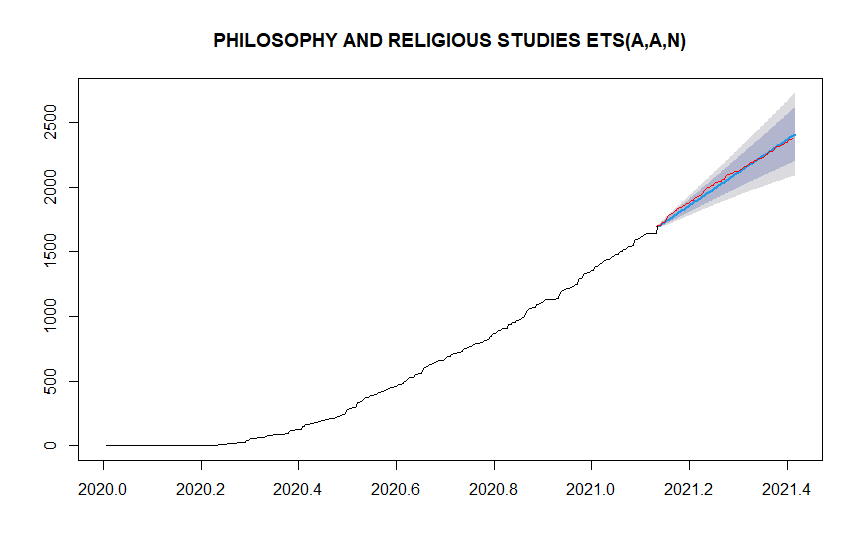 |
| 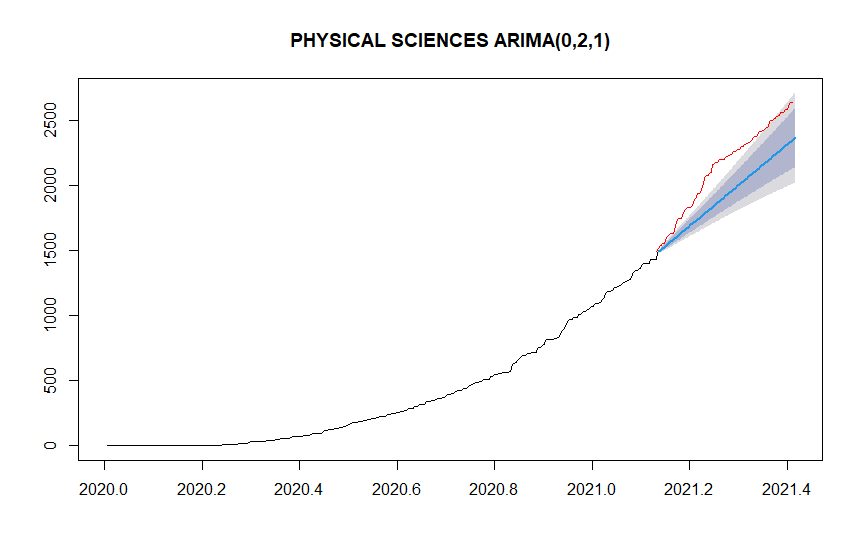 | 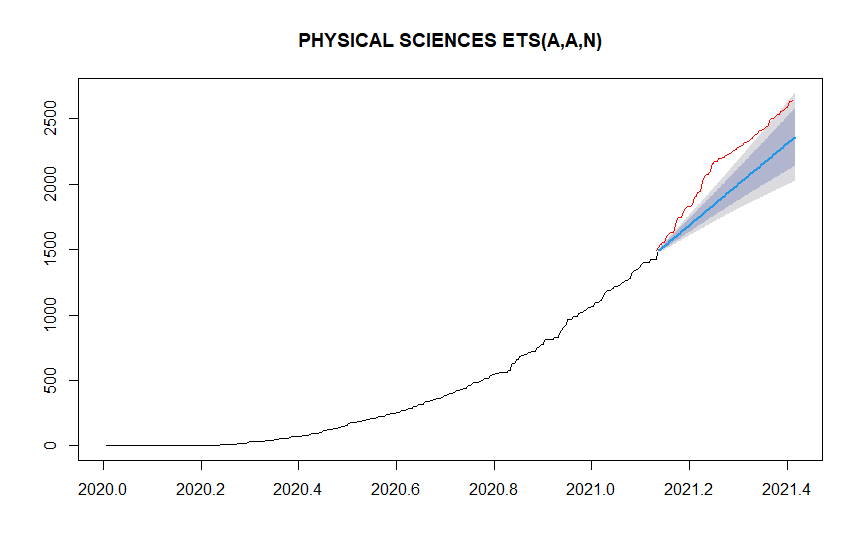 |
| 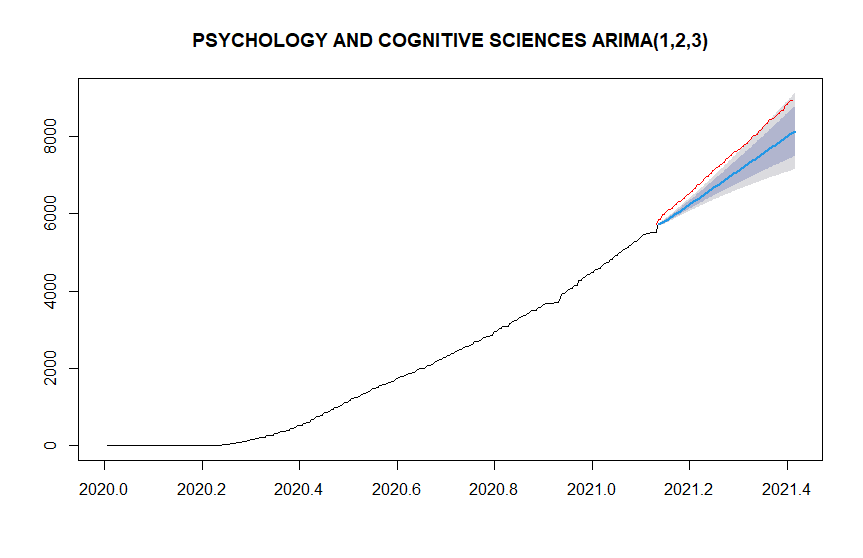 | 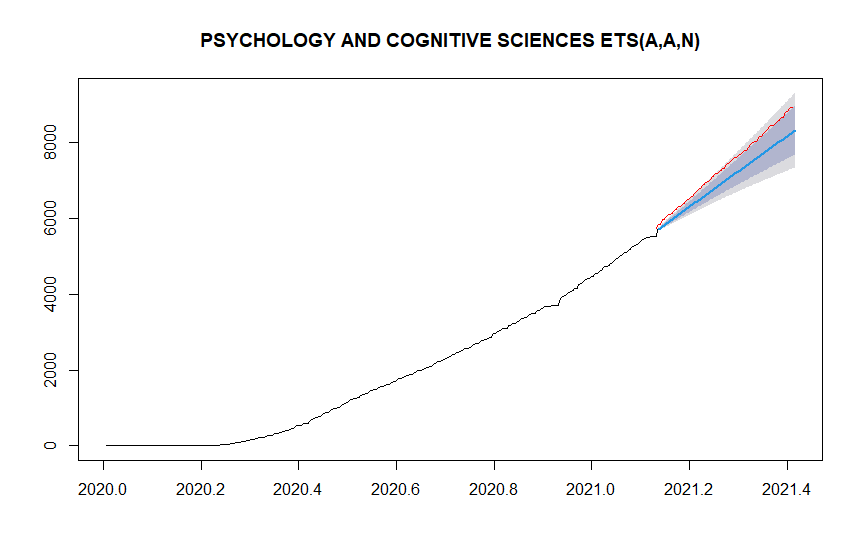 |
| 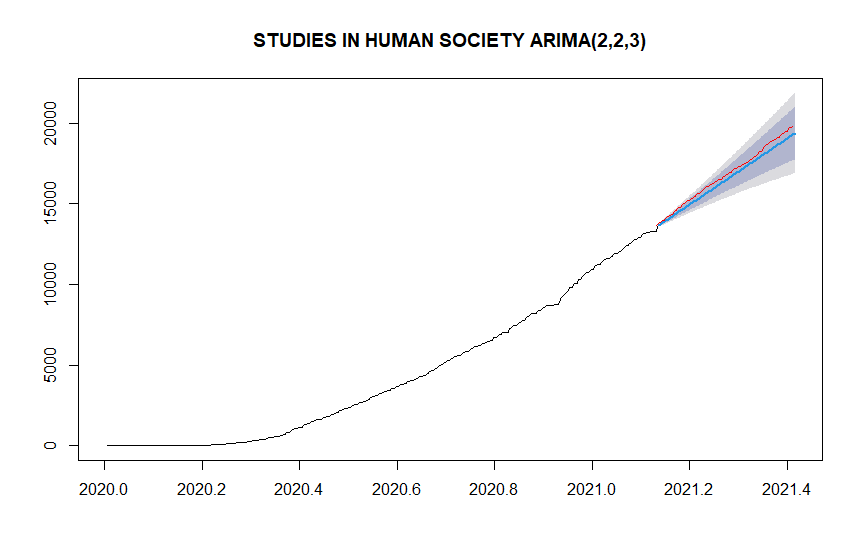 | 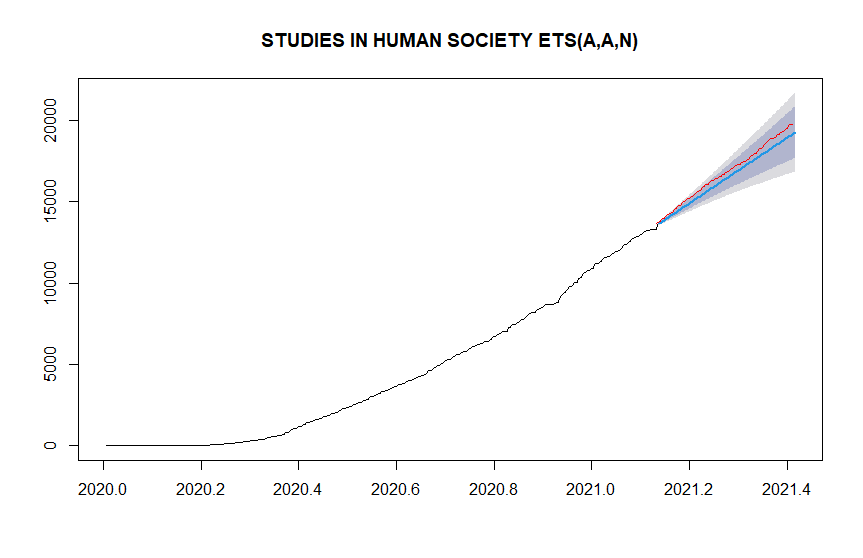 |
| 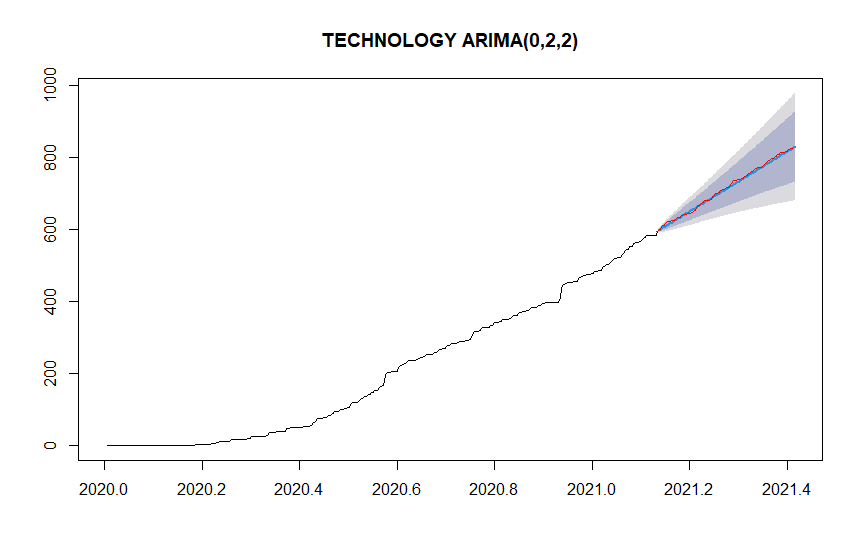 | 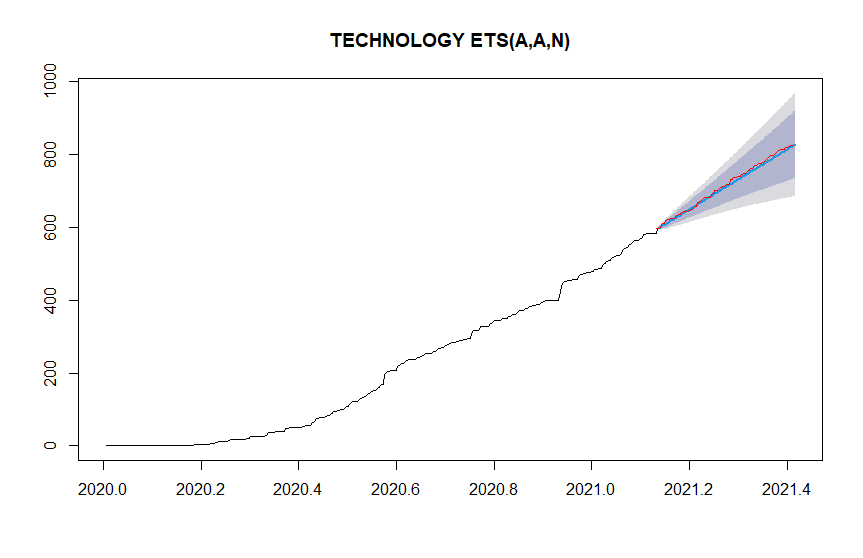 |

1. Corresponding autor: g.f.nane@tudelft.nl [↑](#footnote-ref-1)
